# Early Insights into the Function of KIAA1199, a Markedly Overexpressed Protein in Human Colorectal Tumors

**DOI:** 10.1371/journal.pone.0069473

**Published:** 2013-07-23

**Authors:** Amit Tiwari, Mirjam Schneider, Antonio Fiorino, Ritva Haider, Michal J. Okoniewski, Bernd Roschitzki, Anuli Uzozie, Mirco Menigatti, Josef Jiricny, Giancarlo Marra

**Affiliations:** 1 Institute of Molecular Cancer Research, University of Zurich, Zurich, Switzerland; 2 Department of Preventive and Predictive Medicine, Fondazione IRCCS Istituto Nazionale dei Tumori, Milano, Italy; 3 Functional Genomics Center of the ETH and University of Zurich, Zurich, Switzerland; Vanderbilt University, United States of America

## Abstract

We previously reported that the expression of *KIAA1199* in human colorectal tumors (benign and malignant) is markedly higher than that in the normal colonic mucosa. In this study, we investigated the functions of the protein encoded by this gene, which are thus far unknown. Immunostaining studies were used to reveal its subcellular localization, and proteomic and gene expression experiments were conducted to identify proteins that might interact with KIAA1199 and molecular pathways in which it might play roles. Using colon cancer cell lines, we showed that both endogenous and ectopically expressed KIAA1199 is secreted into the extracellular environment. In the cells, it was found mainly in the perinuclear space (probably the ER) and cell membrane. Both cellular compartments were also over-represented in lists of proteins identified by mass spectrometry as putative KIAA1199 interactors and/or proteins encoded by genes whose transcription was significantly changed by KIAA1199 expression. These proteomic and transcriptomic datasets concordantly link KIAA1199 to several genes/proteins and molecular pathways, including ER processes like protein binding, transport, and folding; and Ca^2+^, G-protein, ephrin, and Wnt signaling. Immunoprecipitation experiments confirmed KIAA1199’s interaction with the cell-membrane receptor *e*phrin A2 and with the ER receptor ITPR3, a key player in Ca^2+^ signaling. By modulating Ca^2+^ signaling, KIAA1199 could affect different branches of the Wnt network. Our findings suggest it may negatively regulate the Wnt/CTNNB1 signaling, and its expression is associated with decreased cell proliferation and invasiveness.

## Introduction

The function of the protein encoded by *KIAA1199* is currently unknown, but our previous studies have documented marked overexpression of this gene–at both the mRNA and protein levels–in almost all colorectal tumors (benign and malignant) [1], [2]. More recently, similar upregulation has also been reported in a large series of colorectal cancers [3] and in gastric cancers [4]. On the basis of our findings (gene expression data, protein expression patterns in colorectal tissues, the results of *in silico* analysis), we have suggested that *KIAA1199* might be a Wnt target gene. It appears to encode a secreted membrane/cytoplasmic protein that is structurally similar to several other eukaryotic proteins, including transmembrane protein 2, polyductin, and fibrocystin L, all of which are large receptor proteins with an N-terminal signal peptide or a single transmembrane helix and short cytoplasmic tail [1].

These findings raise interesting possibilities for the development of a new molecular marker for detecting gastrointestinal neoplasms. *KIAA1199* mRNA has been detected in the plasma of almost 80% of individuals with colorectal adenomas or cancers [5]. Unfortunately, it was also identified in 30% of tumor-free controls, so its ultimate diagnostic value remains to be seen. Attempts have also been made to explore the prognostic significance of *KIAA1199* mRNA or protein expression levels in colorectal [3] and gastric [4] cancers and in mesotheliomas [6], but in most cases the study cohorts were too small to allow any meaningful conclusions to be drawn. In the meantime, little or no progress has been made toward identifying KIAA1199’s role(s) in normal and neoplastic cells. The present study represents a first step toward the filling of this gap.

## Materials and Methods

### Cell Lines

Human SW480 colon cancer cells from the Cancer Network Zurich cell line repository were used for most of the experiments conducted in this study. Their authenticity was verified by microsatellite analysis performed in January 2012 at Microsynth AG, Switzerland. The cells were cultured in RPMI 1640 medium (Invitrogen) supplemented with 10% fetal calf serum (Gibco), 100 IU penicillin, and 100 µg/ml streptomycin.

Clones that constitutively express KIAA1199 were obtained by Lipofectamine 2000 (Invitrogen)-mediated transfection of SW480 cells with the pcDNA3.2V5DEST vector containing *KIAA1199* ORF (described below). The clones were selected and maintained in medium containing G418 (0.4 mg/ml) (Gibco).

Inducible KIAA1199 expression was achieved with a two-step procedure. First, SW480 cells were transfected with the pcDNA6TR vector (Invitrogen) and the tetracycline repressor-expressing cells selected with 7 µg/ml blasticidin S (InvivoGen). The blasticidin-resistant cells were then transfected with the pT-Rex-DEST30-KIAA1199 construct (described below) and selected with 0.4 mg/ml G418. The growth medium used for these cells was supplemented with 10% Tet-system-approved fetal calf serum (Biochrom). For induction of KIAA1199 expression, doxycycline (Clontech) was used at concentrations of 50–200 ng/ml, which have been shown to have no effects on transcript levels measured with microarrays [7].

### Cloning of Full-length KIAA1199

Full-length *KIAA1199* was PCR-amplified from LS180 cell cDNA with Phusion Hot-Start High Fidelity DNA Polymerase (Finnzyme) and the following primers: forward 5′-GGA TAT GGT ACC ACA CTG CCA GGA TGG GAG CTG-3′, reverse 5′-GAT ATC TCG AGC TAC AAC TTC TTC TTC TTC ACC ACA GGG ATG-3′. The amplified *KIAA1199* open reading frame was then inserted into the pENTR11 vector (Invitrogen) with the aid of *Kpn*I*/Xho*I restriction enzymes, and an LR recombination reaction (Gateway, Invitrogen) was carried out to clone it into the constitutive expression vector pcDNA3.2V5DEST (with or without a C-terminus V5 tag) and into the Tet-on inducible vector pT-Rex-DEST30 (untagged and C-terminus GFP-tagged proteins). The C-terminus was chosen for tagging since KIAA1199 contains a signal peptide at its N-terminus.

### Whole-cell Lysates and Conditioned Medium Concentrates

Cells were trypsinized, washed with PBS, and placed in cold single-detergent lysis buffer (1% NP-40, 350 mM NaCl, 50 mM Tris-Cl, 1 mM EDTA, 1X Complete EDTA-free Protease Inhibitor Cocktail [Roche], and 1 mM PMSF) for 60 minutes. After 45 min of centrifugation (20,800 *g* at 4°C), the clarified lysates were aliquoted and snap-frozen in liquid nitrogen.

When cells reached ∼80% confluence, complete medium was removed, and the cells were washed with PBS and cultured in serum-free medium for 8 hours. The medium was then collected and supplemented with 1 mM PMSF and 1X Complete EDTA-free. Floating cells were spun down at 100 *g* for 5 minutes, and the clarified medium was concentrated by centrifugation at 3000 *g* with Amicon Ultra centricons (10 kDa NMWL).

### Subcellular Fractionation

Cells were harvested by trypsinization, washed with PBS, and centrifuged. The pellets were resuspended (∼10^8^ cells/ml) in cold hypotonic buffer (200 mM Hepes pH 7.9, 50 mM KCl, 15 mM MgCl_2_, 1 mM PMSF and 1X Complete EDTA-free, 1 mM DTT). Cells were then transferred to a Dounce homogenizer, left to swell on ice for 5 minutes, and lysed with a B type pestle until microscopic examination revealed that most nuclei were trypan blue-positive but still intact. The nuclei were then spun down (2000 *g* for 10 minutes), and the supernatant (i.e., the cytoplasmic extract) was clarified (12,000 *g*), aliquoted, and frozen. The nuclei were then resuspended in half the packed nuclear volume of low-salt buffer (20 mM Hepes pH 7.9, 25% glycerol, 1.5 mM MgCl_2_, 20 m M KCl, 0.2 mM EDTA, 0.5 mM PMSF, and 0.5 mM DTT) and stirred. Half the packed nuclear volume of high-salt buffer was then added (0.6 M KCl in low-salt buffer) drop-wise. The suspension was stirred for 30 min and centrifuged at 20,000 *g* to extract nuclear proteins. The pellet was resuspended in single-detergent lysis buffer (described above), incubated for 30 minutes, and centrifuged at 20,800 *g*. The supernatant (referred to hereafter as the ER-nuclear membrane-chromatin fraction) was aliquoted and snap-frozen. All steps were carried out on ice in the cold room.

### Cell-membrane Extract

Cells grown in 15-cm dishes were washed 3 times with PBS and lysed in 1 ml/dish lysis buffer (300 mM mannitol, 20 mM Hepes-Tris pH 7.4, 2 mM EDTA, 1 mM PMSF, 1X Complete EDTA-free). The cell suspension was homogenized by syringing with a 25G needle, and lysis was verified under the microscope with trypan blue staining. The lysate was centrifuged for 10 min at 100 *g* (4°C) to eliminate cellular debris and then ultracentrifuged (287,660 *g*) at 4°C for 45 minutes. The supernatant was discarded. The membrane pellet was homogenized by syringing with a 25G needle and suspended in a resuspension buffer (300 mM Mannitol, 20 mM Hepes-Tris pH 7.4, 2 mM EDTA, 0.1% Triton X-100, 0.5% Na-deoxycholate, 1 mM PMSF, 1X Complete EDTA-free) to obtain the cell membrane extract.

### Antibodies

The following primary antibodies were used: rabbit polyclonal anti-KIAA1199 (SDIX, no. 4575.00.02); mouse monoclonal anti-V5 tag antibodies (Abcam, no. ab27671 for Western blotting and immunohistochemistry; Invitrogen, no. R960-25 for immunoprecipitation); mouse monoclonal anti-TUBB (beta-tubulin) (Santa Cruz, no. sc-5274); rabbit polyclonal anti-TFIIH (Santa Cruz, no. sc-293); mouse monoclonal anti-CDH1 (E-cadherin) (BD Transduction Laboratories, no. 610181); mouse monoclonal anti-CTNNB1 (beta-catenin) (Dako, no. M3539); mouse monoclonal anti-active CTNNB1 (Millipore, no. 05-665), which specifically detects beta-catenin dephosphorylated at residues S37 and T41; mouse monoclonal anti-ITPR3 (BD Transduction Laboratories, no. 610312); mouse monoclonal anti-EPHA2 (Millipore, no. 05-480); mouse monoclonal anti-NME2 (Abnova, no. H00004831-M06); rabbit polyclonal anti-UBR5 (Bethyl Laboratories, no. A300-573A); mouse monoclonal anti-CUL1 (Invitrogen, no. 32-2400); mouse monoclonal anti-LGALS3 (Novocastra, no. NCL-GAL3); rabbit polyclonal anti-CANX (calnexin), a kind gift from Dr. Ari Helenius of ETH Zurich; and rabbit polyclonal anti-MYC (Santa Cruz, no. sc-764).

### Immunoprecipitation and Mass Spectrometry

Cells were washed with cold PBS and scraped with a rubber policeman into immunoprecipitation (IP) lysis buffer (150 mM NaCl; 1% NP-40; 50 mM Tris-HCl, pH = 8.0, 1 mM PMSF, 1 mM Na_3_VO_4_, 1 mM NaF, 1X Complete EDTA-free). The lysate was passed through a 25G needle 10 times, incubated on ice for 1 hour, and cleared by 45 min of centrifugation at 20,800 *g*. A 10-microgram extract was pre-cleared by incubation for 2 hours with Protein G Sepharose beads (GE Healthcare) for mouse monoclonal antibodies or Protein A/G Agarose beads (Santa Cruz) for rabbit polyclonal antibodies. The extract was then incubated with 5 µg of primary antibody (overnight, 4°C) and subsequently with Protein-G or A/G beads (1 h). The beads were then washed 3 times with IP lysis buffer and boiled with 1X SDS-bromophenol blue dye at 95°C. After 2 minutes of centrifugation at 2700 *g*, the supernatant was collected in a new tube. The beads (i.e., the pellet) were boiled again with 1X SDS-bromophenol blue dye at 95°C and centrifuged at 2700 *g*. The supernatant thus obtained was combined with the previous one.

Immunoprecipitated proteins were separated according to molecular mass on NuPAGE Novex 4–12% Bis-Tris gels (Invitrogen) and stained with Roti®-Blue Colloidal Coomassie (Carl Roth GmbH). The gel lane was cut into 35 slices, and each was destained. After in-gel digestion with trypsin (Promega), the peptide-containing supernatants were purified via C18–ZipTip pipette tips (Millipore). The tryptic peptides were separated on a reverse-phase tip column (75 µm ×80 mm) packed with silica-based, reverse-phase C_18_ material (3 µm, 200 Å, AQ, Bischoff GmbH) using a linear gradient of 1% to 35% acetonitrile in 60 min. The eluted peptides were analyzed on an LTQ-Orbitrap mass spectrometer or an LTQ-ICR-FT-Ultra mass spectrometer (Thermo Fisher Scientific) in the data-dependent acquisition mode. Mass spectrometry (MS) data were searched against the Swissprot database (release 2011) using the Mascot search algorithm (Matrix Science) and loaded into Scaffold (Proteome Software) for further analysis. Protein assignments were accepted if peptide and protein probability scores (generated by Peptide Prophet and Protein Prophet algorithms) were ≥95% and ≥80%, respectively [8], [9].

### Microarray Analysis

Using the RNeasy Mini Kit (Qiagen), we extracted total RNA from cells in which KIAA1199 expression had been induced by exposure to doxycycline (48 hours) and from uninduced control cells (3 independent experiments). Methods used for quality assessment of RNA, cDNA generation, hybridization of fragmented and biotin-labeled DNA to Affymetrix GeneChip Human Exon 1.0 ST array, and data processing have been described in detail elsewhere [10]. Gene expression data obtained with 21,980 transcript clusters were analyzed with the Partek Genomics Suite (Partek). The original microarray data have been deposited in the National Center for Biotechnology Information’s Gene Expression Omnibus (GEO, http://www.ncbi.nlm.nih.gov/geo/) and are accessible through GEO Series accession number GSE39575.

### Immunofluorescence, Immunocytochemistry, and Western Blot

For immunofluorescence experiments, cells grown on glass cover-slips were washed with PBS, fixed at room temperature with 4% paraformaldehyde (20 minutes), permeabilized with 0.2% Triton-X 100 (5 minutes), and blocked with 5 mg/ml BSA in PBS (30 minutes). They were then incubated with primary antibody (overnight, 4°C) and then with secondary antibodies (1 hour, 37°C). The cells were covered with DAPI-Vectashield mounting medium (Vector Laboratories), and images were captured on an epifluorescence microscope (Leica) equipped with Leica Application Suite V3.3.0 software. Exposure settings were adjusted in each experiment so that no fluorescence signals were observed in negative control slides (i.e., those incubated without primary antibodies).

Immunocytochemistry and Western blotting were performed as previously described [10], [11].

### Luciferase Reporter Assay

CTNNB1 (beta-catenin)-dependent transcriptional activity was studied in HEK293 cells transfected with the TOPflash or FOPflash reporter vectors (Upstate Biotechnology), which contain a multimeric consensus TCF/LEF binding site and an inactive mutated homologue of this site, respectively. Briefly, cells were seeded in 24-well plates (2×10^4^ cells/well), cultured for 16 hours, and transfected in triplicate (via Lipofectamine LTX, Invitrogen) with a mixture of 75 ng reporter vector, 20 ng pCMVbeta-Gal co-reporter, and 50 ng pcDNA3-CTNNB1 (wild-type or constitutively active CTNNB1^T41A^ kindly provided by M.A. Buendia of the Institut Pasteur in Paris), plus 120 ng of either mock, KIAA1199, or TCF4-DN plasmid (this latter from Hans Clevers, Hubrecht Institute, Utrecht, NL). Medium was replaced 8 hours post-transfection, and luciferase activity was assayed 48 h later with the Promega Luciferase assay kit (Promega). Results for each sample were normalized to beta-galactosidase activity (High-sensitivity beta-galactosidase assay kit, Stratagene). For each construct combination, the TCF/LEF-specific transactivation was quantified by dividing the TOPflash luciferase activation by the average FOPflash activity. Luciferase reporter assays were also performed in SW480 Cl.18 cells.

### Proliferation, Colony Formation, Transwell Invasion and Migration Assays

Cells were seeded in duplicate in 6-well plates (5000 cells/well) and grown under standard conditions. At each time point, cells were trypsinized, stained with trypan blue, and counted with a hemocytometer.

For colony formation assays, cells were seeded in triplicate in 100-mm dishes (1000, 500, and 100 cells/dish). After 11 days of growth, the cells were fixed (10 minutes in methanol/PBS, 1∶1), washed in pure methanol (2 minutes), and stained with 20 mg/ml of Crystal Violet Certistain (Merck). Colonies containing >50 cells were identified by manual counts under the microscope.

For invasion assays, 8-microm transwell permeable supports (BD Biosciences) were coated with 50 µl of 0.5 µg/µl Matrigel (BD Biosciences), allowed to dry for 3 hours at room temperature, and rehydrated with 300 µl RPMI medium for 1 hour at 37°C. The upper chamber was seeded (in triplicate) with 200,000 cells; the lower chamber was filled with medium containing 500 µl of 20% fetal calf serum (as a chemoattractant). Forty-eight hours later, the insert was cleaned thoroughly with a cotton swab to remove any cells that had not passed through the membrane. It was then placed in 2% formaldehyde (freshly prepared in PBS) to fix the cells (10 minutes) and washed 3 times with PBS. DNA was stained with DAPI (10 minutes), and 20 randomly selected fields were imaged with an Olympus IX81 microscope equipped with Cell^∧^R software (Olympus, Japan). Stained nuclei were counted with the Count tool of the Adobe Photoshop program (Adobe).

Cell migration was comparatively assessed in KIAA1199 expressing clones and SW480 Dest (controls) using the in vitro scratch test, as described elsewhere [12].

### Statistical Analysis

For microarray experiments, p values were generated with a paired, two-tailed t test performed in the Partek Genomics Suite and the results subjected to Benjamini-Hochberg correction. To select genes whose mRNA levels changed upon KIAA1199 expression, we used an unadjusted p value threshold of 0.025, the equivalent of a Benjamini-Hochberg adjusted p value of 0.55.

For other experiments reported in this study, two-tailed p values were calculated with paired or unpaired t tests performed with the GraphPad Prism 5.0 statistical software package.

## Results

Figure S1A shows *KIAA1199* mRNA expression data from our previous study on benign and malignant colorectal tumor tissues, normal colorectal mucosal samples, and 8 colon cancer cell lines [1]. The widely variable expression levels observed in these cell lines were also seen in Western blot experiments in the present study (Figure S1B). They probably reflect epigenetic changes at the *KIAA1199* promoter, as recently described in human breast cancer cells [13].

**Figure 1:**
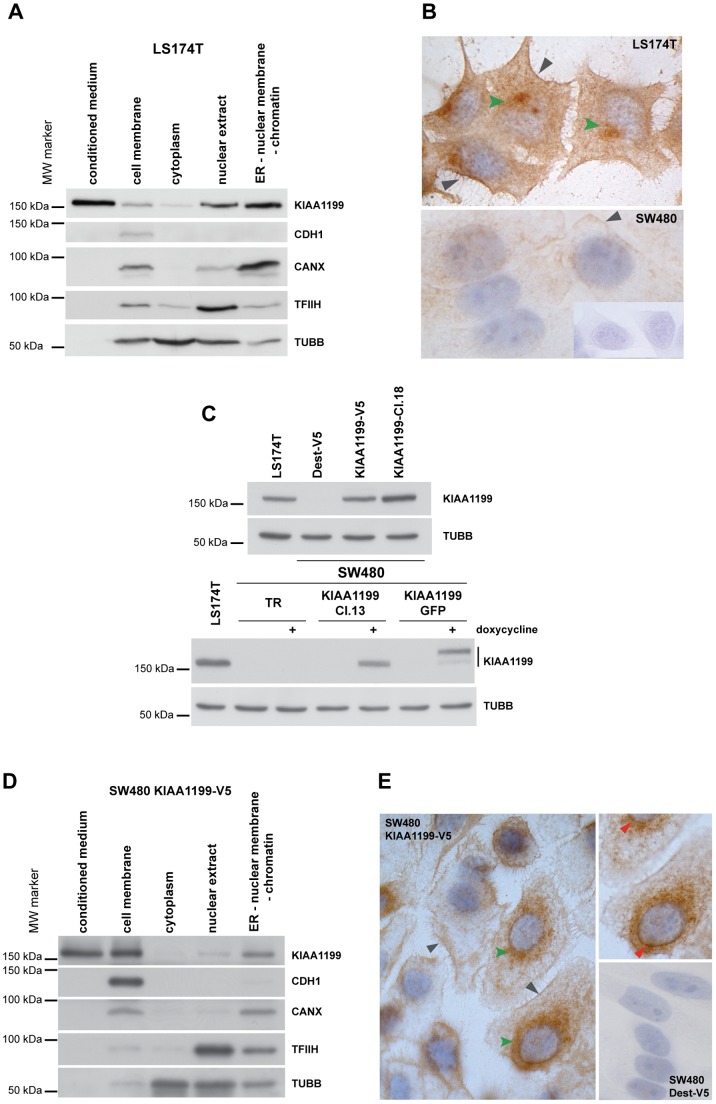
Expression and localization of KIAA1199. **A.** Western blots showing KIAA1199 protein levels in distinct subcellular compartments of LS174T cells and in conditioned medium. CDH1 (E-cadherin), CANX (calnexin), TUBB, and TFIIH were used as cell-membrane, ER, cytoplasmic, and nuclear markers, respectively. **B.** Immunocytochemical localization of KIAA1199 in LS174T (upper panel) and SW480 cells (lower panel). Gray arrowheads: cell membrane expression; green arrowheads: perinuclear expression. Inset: negative control (immunostaining without primary antibody). **C.** Constitutive and inducible expression of KIAA1199 in transfected SW480 cells. Top: SW480 clones expressing KIAA1199 constitutively. Dest-V5, cells transfected with empty vector pcDNA3.2V5DEST; KIAA1199-V5, cells expressing KIAA1199-V5 tagged at C-terminus; KIAA1199-Cl.18, cells expressing untagged KIAA1199. Bottom: SW480 clones expressing KIAA1199 upon doxycycline induction. TR, tetracycline-repressor-expressing control cells; KIAA1199-Cl.13, cells expressing KIAA1199 only in the presence of doxycycline; KIAA1199-GFP, cells expressing C-terminal GFP-tagged KIAA1199 only in the presence of doxycycline. The total cell extract of LS174T cells (endogenous KIAA1199 expressors) was used as a positive control; TUBB was used as loading control. **D.** Subcellular localization of KIAA1199 in SW480 KIAA1199-V5 cells. CDH1, CANX, TUBB, and TFIIH were used as cell-membrane, ER, cytoplasmic, and nuclear markers, respectively. **E.** Immunocytochemical staining of SW480 KIAA1199-V5 cells shows KIAA1199 in the cell membrane (gray arrowheads) and perinuclear space (green arrowheads). Upper right inset: Fine focusing clearly reveals staining of the nuclear membrane (red arrowheads). Lower right inset: Negative control (SW480 Dest-V5 immunostained with V5-tag-specific antibody).

The LS174T cell line, which displayed the highest expression at both the mRNA and protein levels, was used to investigate the subcellular localization of endogenous KIAA1199 protein. Collectively, our Western blot (Figure 1A) and immunocytochemistry data (Figure 1B) indicated that the KIAA1199 in these cells is found mainly in the perinuclear space (presumably the ER, including the outer nuclear membrane and ER tubules) and cell membrane–a pattern similar to that found in normal colorectal epithelial cells [1]. The protein also appeared to be abundantly secreted (Figure 1A). Treatment of LS174T protein extracts with Peptide N-glycosidase F (PNGaseF), which cleaves asparagine-linked (N-linked) oligosaccharides from glycoproteins, increased KIAA1199 mobility in Western blotting (Figure S2A).

Restoration of KIAA1199 expression was investigated in SW480 cells, where KIAA1199 expression is minimal-absent (Figure 1B and Figure S1). We established SW480 clones that expressed tagged (C-terminal V5 or GFP) or untagged forms of KIAA1199, constitutively (KIAA1199-V5 and KIAA1199-Cl.18) or upon doxycycline induction (KIAA1199-Cl.13 and KIAA1199-GFP) (Figure 1C). The subcellular KIAA1199 distribution patterns in all these clones were virtually identical to that of endogenous KIAA1199 in LS174T cells (see example in Figures 1D and 1E), and all secreted the exogenously expressed protein **(**tagged and untagged forms) into the conditioned media (Figure 1D).

**Figure 2:**
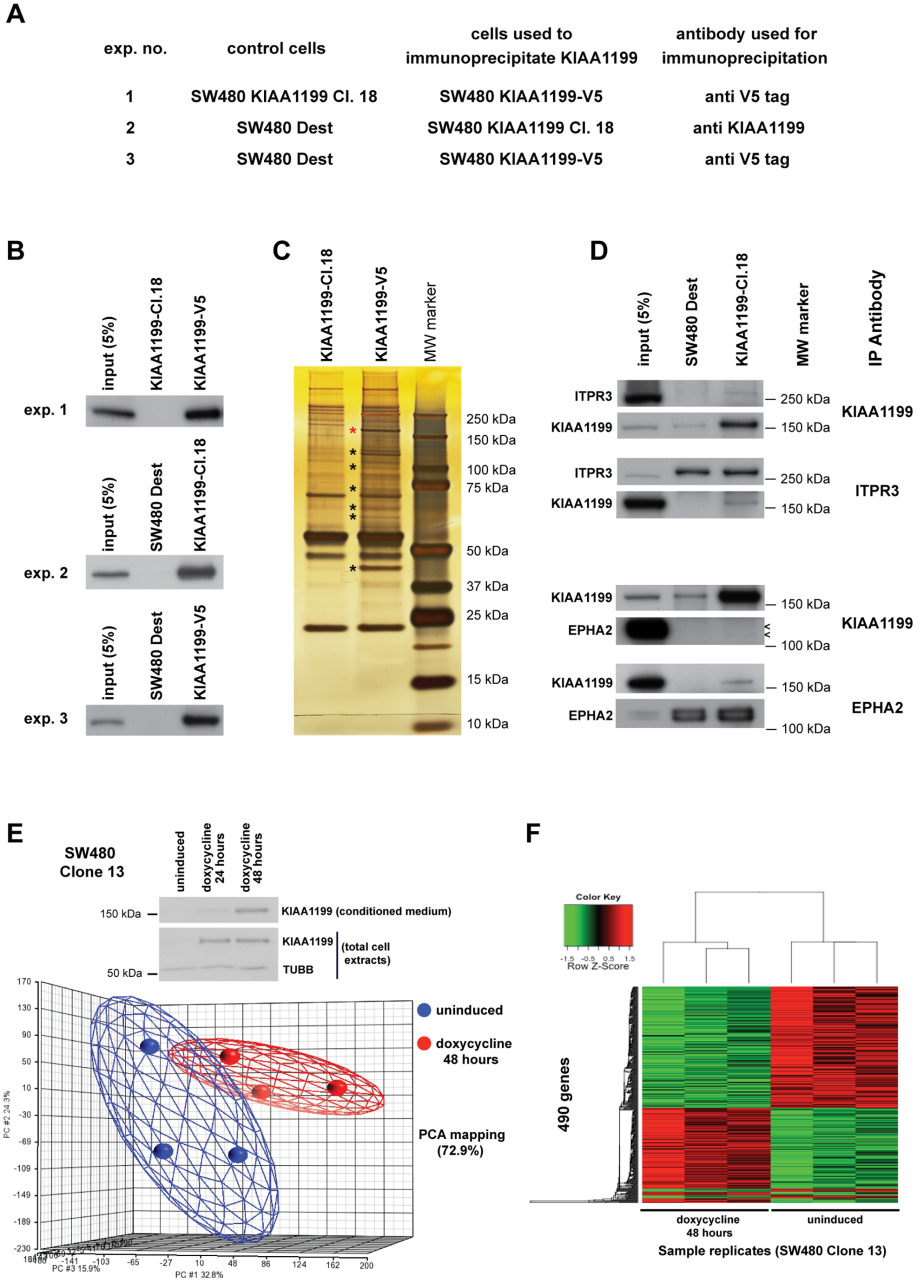
KIAA1199 interactome and gene expression changes associated with KIAA1199 expression in SW480 cells. **A.** The table shows cell lines and antibodies used in the three immunoprecipitation experiments performed to identify proteins that interact with KIAA1199. **B.** Representative Western blots showing the results of the immunoprecipitation experiments summarized in the table. Input: exp.1 and 3, KIAA1199-V5 extract; exp.2, KIAA1199-Cl.18 extract. **C.** Silver-stained gel showing unique bands present only in the KIAA1199 immunoprecipitate (black asterisk) following IP with antibodies against the V5 tag. (The immunoprecipitated KIAA1199 is indicated with a red asterisk.) **D.** Upper four strips: Western blot showing co-immunoprecipitation of ITPR3 with anti-KIAA1199 antibody and reciprocal co-immunoprecipitation of KIAA1199 with anti-ITPR3 antibody. Lower four strips: Western blot showing co-immunoprecipitation of EPHA2 with anti-KIAA1199 antibody (arrowheads) and reciprocal co-immunoprecipitation of KIAA1199 with anti-EPHA2 antibody. Input: KIAA1199-Cl.18 extract. The weak interactions of these two proteins with KIAA1199 might be related to the fact that IP was performed using extracts from cells in which these two proteins were endogenously expressed (instead of overexpressed). **E.** Top: Western blot showing KIAA1199 protein levels in SW480 Clone 13 total cell extracts and in conditioned medium before and after doxycycline induction. TUBB: loading control. Bottom: Principal component analysis (PCA) of log_2_ *KIAA1199* mRNA expression intensity values. The plot of PCA scores for the six samples 48 h after doxycycline induction shows clear separation of replicates with (n = 3) vs. without (n = 3) KIAA1199 expression. The first three principal components explain 72.9% of the total variance. **F.** Heatmap showing expression of the 490 differentially expressed genes (*y* axis) before and after doxycycline-induced KIAA1199 expression in three replicate experiments. (Green: upregulated; red: downregulated in KIAA1199-expressing cells; p value <0.025, and fold change >1.2).

The KIAA1199-V5 and KIAA1199-Cl.18 clones, which displayed relatively high constitutive expression of KIAA1199 (Figure 1C), were used in IP/MS experiments designed to identify proteins that might interact with KIAA1199. Figure 2A to 2C show the cells and antibodies used in each of the 3 independent IP experiments and an example of the silver staining band pattern of the KIAA1199 immunoprecipitate. Eighty-nine proteins (Table S1) were identified as putative KIAA1199 interactors on the basis of MS analysis of co-immunoprecipitates. All met the following criteria in at least 2 of the 3 experiments: 1) their peptides were present in the KIAA1199 immunoprecipitates and absent in control immunoprecipitates described in Figure 2A; or 2) their presence in the KIAA1199 immunoprecipitates was observed at least 4 times more frequently than in the control immunoprecipitates. Validation IP experiments were performed for several of these proteins (CANX, CUL1, EPHA2, ITPR3, LGALS3, NME2, and UBR5; see antibodies used in *Material and Methods*). ITPR3 and EPHA2 emerged as *bona fide* KIAA1199 interactors (Figure 2D and Figure S3). IP is often performed with extracts from cells in which the proteins of interest are overexpressed. The extracts used in our experiments contain only endogenously expressed ITPR3 and EPHA2, and this may partly account for the relatively weak interaction between these proteins and KIAA1199.

We also investigated gene expression changes 48 h after doxycycline-mediated induction of KIAA1199 expression in KIAA1199-Cl.13 cells. Use of this isogenic cellular system allowed prompt detection of changes that occurred shortly after KIAA1199 expression was switched on. (The assay time point was chosen on the basis of the Western blot data shown in Figure 2E). In principal component analysis, KIAA1199-expressing cells were clearly distinguished from the uninduced cells (Figure 2E, three independent experiments), and expression of the protein was associated with significant changes in the transcript-level expression of 490 genes (Figure 2F, Table S2). Fold changes were generally low, pointing to a moderate effect of KIAA1199 expression on the transcriptome of these cells. However, as reported in *Material and Methods*, the genes listed in Table S2 were selected using a low level of stringency. This approach was associated with a high false discovery rate, but it increased our chance of selecting genes that were also among the putative KIAA1199 interactors identified in our MS experiments. In fact, EPHA2 and ITPR3 were included in both lists although their mRNA and protein levels were only modestly changed upon KIAA1199 expression (Figure S4). The two lists were then interrogated with GeneGO MetaCore software (Thomson Reuters) to identify any gene ontology categories or interactomes that might be over-represented therein (Table 1). The results of these analyses are discussed extensively below (see *Discussion*).

**Table 1:** Gene ontologies (GO) and interactomes over-represented in the lists of putative KIAA1199 interactors and genes whose transcript levels were affected by KIAA1199 expression.

89 putative interactors of KIAA1199 (IP/MS data)		490 genes whose expression changed upon KIAA1199 expression (microarray data)	
Enrichment Ontologies and Interactome	p value	Enrichment Ontologies and Interactome	p value
**GO cellular localization**		**GO cellular localization**	
* Cytoplasm*		* Organelle*	
cytoplasmic part	2.93E-10	organelle part	2.18E-14
cytoplasm	3.28E-10	intracellular organelle	1.10E-13
* Organelle*		intracellular membrane-bounded organelle	5.57E-11
pigment granule	4.02E-11	membrane-enclosed lumen	1.05E-06
membrane-enclosed lumen	2.18E-09	intracellular organelle lumen	1.21E-06
organelle lumen	4.97E-09		
cytoplasmic membrane-bounded vesicle	3.72E-07	* Cytoplasm*	
membrane-bounded organelle	4.79E-07	cytoplasmic part	2.60E-08
ER lumen	3.25E-06	cytoplasm	1.66E-06
* Proteosome complex*	1.08E-06		
**Pathway Maps**		**Pathway Maps**	
* CTFR (Cystic fibrosis transmembrane conductance regulator)* * folding and maturation*	9.61E-05	* Transport*	
		Rab-9 regulation pathway	1.13E-05
* G-protein signaling*		Clathrin-coated vesicle cycle	8.29E-04
G protein-coupled receptors in blood coagulation	1.00E-04	Sorting endosome formation in cystic fibrosis	7.49E-02
S1PR1 signaling pathway (also S1PR2 and S1PR3)	1.95E-04	* Cytoskeleton remodeling*	
G-protein signaling_Regulation of cAMP levels by ACM	2.13E-04	Role of Activin A in cytoskeleton remodeling	1.13E-05
Alpha-2 adrenergic receptor regulation of ion channels	2.52E-04	TGF, WNT and cytoskeletal remodeling	5.75E-05
Alpha-2 adrenergic receptor activation of ERK	7.33E-04	Role of PKA in cytoskeleton reorganisation	3.74E-04
		Regulation of actin cytoskeleton by Rho GTPases	5.32E-03
* Cell adhesion*		* Cell adhesion*	
Chemokines and adhesion	5.03E-04	Histamine H1 receptor signaling in the interruption of cell barrier integrity	6.58E-05
Ephrin signaling	3.24E-03	Ephrin signaling	5.25E-03
Histamine H1 receptor signaling in interruption of cell barrier integrity	3.24E-03	* Transcription*	
ECM remodeling	4.88E-03	Role of heterochromatin protein 1 (HP1) family in transcriptional silencing	3.40E-04
		* Apoptosis and survival*	
* Transcription*		Anti-apoptotic action of Gastrin	5.27E-04
Role of Akt in hypoxia induced HIF1 activation	7.26E-04	BAD phosphorylation	4.09E-03
		* Cell cycle*	
* Immune response*		Initiation of mitosis	5.68E-04
Antigen presentation by MHC class I	8.10E-04	* Role of alpha-6/beta-4 integrins in carcinoma progression*	6.52E-04
CCR4-induced chemotaxis of immune cells	1.38E-03	* Development*	
		SSTR1 in regulation of cell proliferation and migration	1.01E-03
* Glycolysis and gluconeogenesis*	1.20E-03	Endothelin-1/EDNRA signaling	1.38E-03
		FGFR signaling pathway	1.50E-03
* Guanosine/hypoxanthine triphosphate metabolism*	2.92E-03	TGF-beta-dependent induction of EMT via RhoA, PI3K and ILK	5.68E-03
		* G-protein signaling*	
* Role of alpha-6/beta-4 integrins in carcinoma progression*	3.24E-03	G-protein alpha-q signaling cascades	1.86E-03
		S1PR2 receptor signaling	2.08E-03
		* Signal transduction*	
		IP3 signaling	7.12E-03
		Activation of PKC via G-protein coupled receptor	8.78E-03
**Metabolic Networks**		**Metabolic Networks**	
Phosphatidylcholine pathway	2.78E-03	Phosphatidylcholine pathway	1.03E-06
		Phosphatidylinositol-3,4-diphosphate pathway	2.59E-04
**GO Processes**		**GO Processes**	
* Nucleobase-containing small molecule metabolic process*	2.21E-10	Protein transport	9.77E-10
* ER processes*		Establishment of protein localization	1.14E-09
ER unfolded protein response	1.30E-09	Nitrogen compound metabolic process	3.33E-09
Response to ER stress	4.95E-09		
ER-nucleus signaling pathway	4.95E-09		
**GO molecular functions**		**GO molecular functions**	
Protein binding	1.00E-14	Protein binding	6.00E-10
Nucleotide binding	2.63E-07		
**Transcription factors**		**Transcription factors**	
SP1	1.12E-10	HNF4-alpha	7.04E-08
MYC	2.27E-10	MYC	2.66E-06
HNF4-alpha	3.64E-06		
ATF-2/c-Jun	8.77E-06		
**Significant interactions within set**		**Significant interactions within set**	
CTNND1/ITGB4 or TLN1		RPL4/CANX or NMP1	
HSP90B1/LRP1 or HYOU1		PXN/LIMK1	
ITGB4/ITGA6		ROCK1/LIMK1	
LGALS3/CTSD or ITGB4			
LRP1/CTSD or CANX			
TARDBP/H1FX			
TLN1/LRP1 or ITGA6			
ITGA6/CANX			

**Figure 3:**
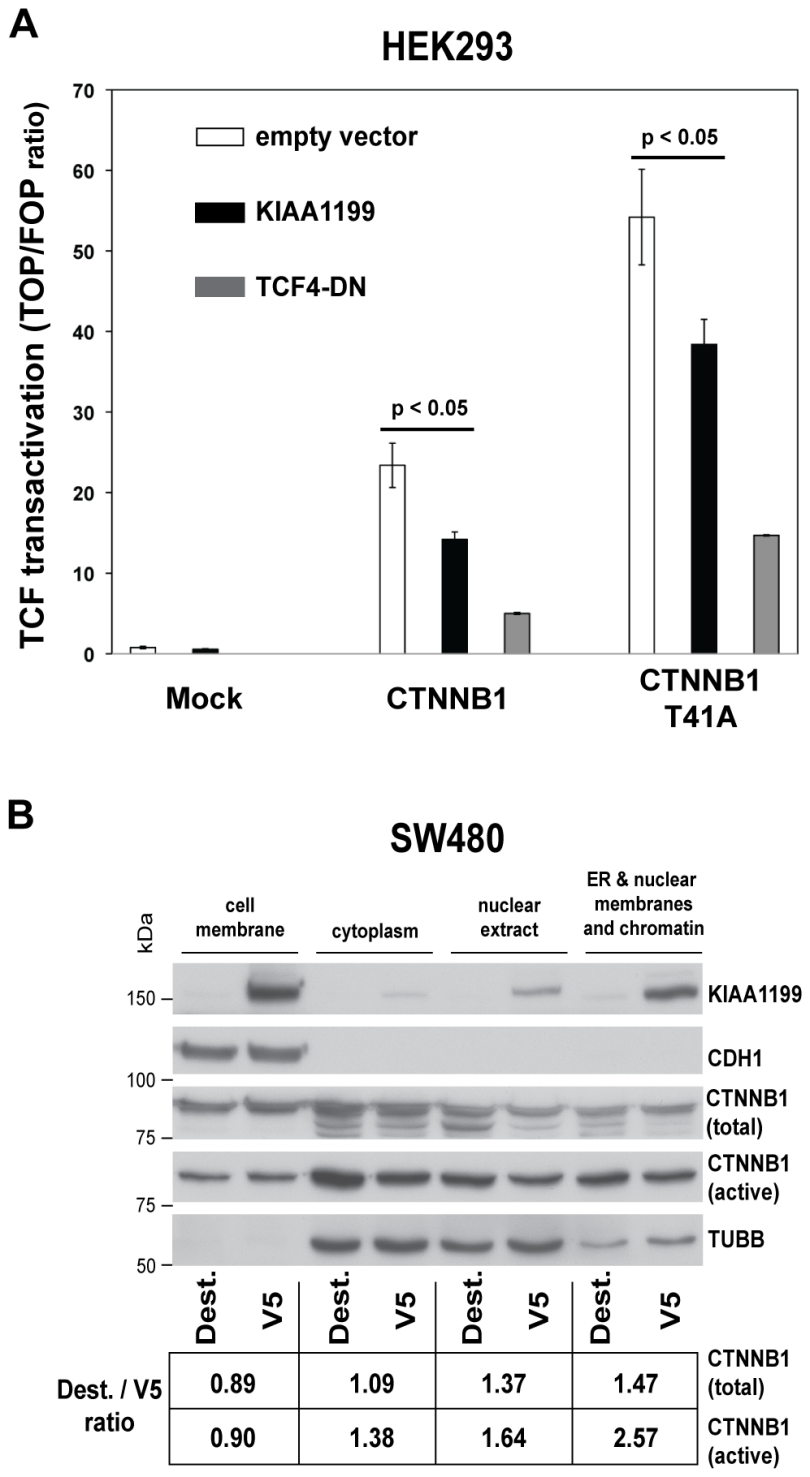
KIAA1199 exerts negative feedback on canonical Wnt signaling. **A.** Ectopic KIAA1199 expression in HEK293 cells inhibits the transcriptional activity elicited by wild-type CTNNB1 or by a constitutively active form of the protein, CTNNB1-T41A. Cells were transiently transfected with either a TOPflash or a FOPflash reporter vector, together with an empty vector or an expression vector for KIAA1199 or TCF4-DN. TOP/FOP ratios are the mean ± SEM of triplicate experiments. **B.** Total and active CTNNB1 levels in SW480 cells expressing KIAA1199 (V5) and in empty vector-transfected (Dest) controls. The table shows fold changes (vs. controls) in the expression of both CTNNB1 forms induced by KIAA1199 expression based on quantification of band intensity relative to that of CDH1 or TUBB in the same lane. TUBB was used as loading control for cytoplasmic and nuclear fractions since it was detectable in these fractions of the SW480-V5 and Dest cell extracts under the same extraction conditions. Furthermore, TUBB has been reported to shuttle to the nucleus ([50] and references herein).

**Figure 4:**
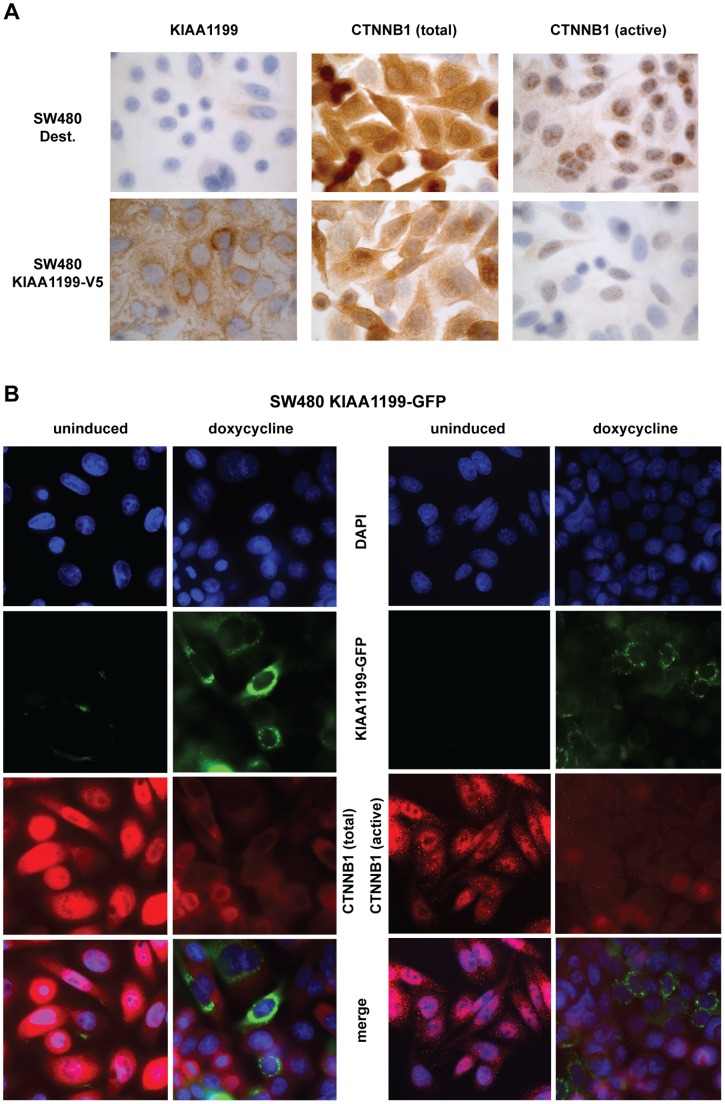
Ectopic expression of KIAA1199 decreases cytoplasmic and nuclear levels of CTNNB1. **A.** Immunocytochemical staining for total and active CTNNB1 in empty vector-transfected SW480 cells (Dest-V5) and SW480 cells expressing KIAA1199-V5 constitutively. The latter cells, presented decreased CTNNB1 levels in the nucleus and cytoplasm. **B.** Similar results were observed in SW480 KIAA1199-GFP cells (immunofluorescence experiments). Although only a fraction of these cells exhibited substantial KIAA1199-GFP expression upon doxycycline induction, the vast majority displayed CTNNB1 depletion (total and active forms) in both the nucleus and cytoplasm, which is probably a paracrine effect of secreted KIAA1199.

We previously showed that *KIAA1199* expression is reduced by induction of a truncated, dominant-negative form of TCF4 in LS174T cells (Figure S2B), suggesting that it might be a novel Wnt signaling target [1]. We now have evidence suggesting that KIAA1199 expression exerts negative feedback on this signaling. TOP/FOPflash reporter assays were used to assess the impact of KIAA1199 expression on transcriptional activity elicited by wild-type CTNNB1 or by CTNNB1^-T41A^, a constitutively active form of the protein with a threonine-for-alanine substitution resulting in a non-phosphorylatable protein at residue 41. In HEK293 cells, which are KIAA1199-nonexpressors (data not shown), ectopic KIAA1199 expression clearly inhibited the transcription evoked by both forms of CTNNB1 (Figure 3A) and significantly reduced the expression of wild-type and constitutively active CTNNB1 (Figure S5A). These effects were less evident in SW480, which are characterized by particularly strong TCF transactivation [14]. Owing in all probability to insufficiently high transfection efficiency, CTNNB1-driven transactivation of the TCF-responsive promoter in SW480 KIAA1199 Cl.18 cells was only modestly (but nonetheless significantly) decreased relative to that observed in SW480 Dest controls (Figure S5B). Consistently, ectopic KIAA1199 expression produced only limited decreases in active CTNNB1 protein levels (i.e., those detected by antibodies recognizing the protein dephosphorylated at residues S37 and T41) and had even fewer effects on total CTNNB1 expression (Figure 3B). This downregulation–in both the nuclear and cytoplasmic compartments–was more evident in immunocytochemical (Figure 4A) and immunofluorescence (Figure 4B) studies performed on KIAA1199-V5 and KIAA1199-GFP clones, respectively. It appears to be a post-transcriptional effect since our microarray experiments revealed no decrease in *CTNNB1* mRNA levels. (Correlation between KIAA1199 and CTNNB1 protein expression patterns in neoplastic tissues could not be assessed since all tumors invariably showed overexpression of KIAA1199 [1].).

**Figure 5:**
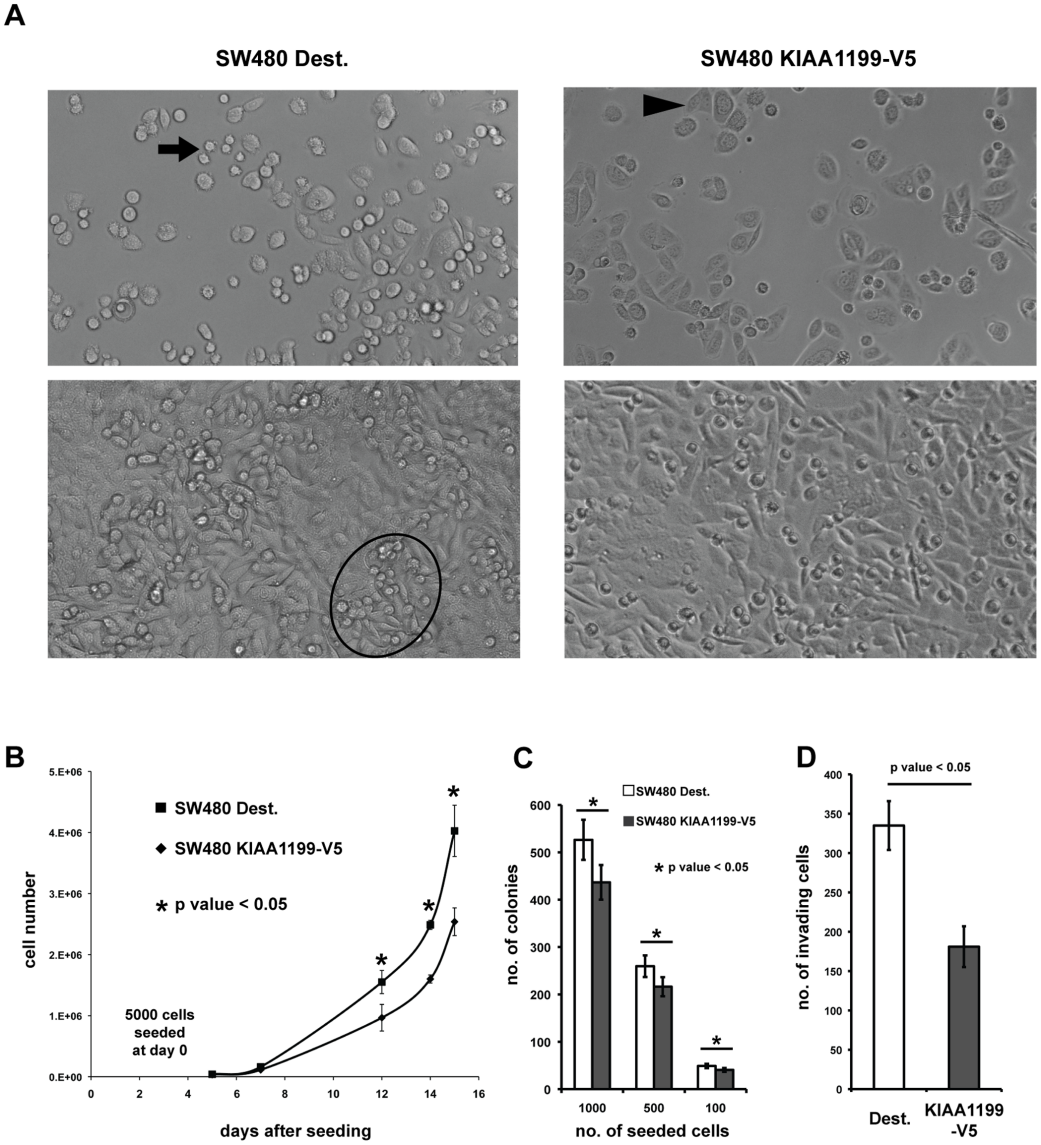
Effect of ectopic constitutive KIAA1199 expression on SW480 cell morphology, proliferation, and invasiveness. SW480 KIAA1199-V5 cells were compared with SW480 Dest-V5 cells (empty vector controls). Each cell phenotype shown in this figure is based on the results of at least three independent experiments. Mean values ± SEM are reported. **A.** Morphology of control (left) and KIAA1199-V5-expressing cells (right) at low (top) and high (bottom) confluence. Control SW480 Dest cells, like the parental SW480 line [15], [16], tended to be round (arrow), and confluent cultures were characterized by clusters of piled-up cells (ellipse). In contrast, KIAA1199-V5-expressing cells were flatter with a more epithelial-like aspect (arrowhead). Compared with controls, KIAA1199-V5-expressing cells also displayed: **B.** diminished proliferation rates; **C.** reduced colony formation; and **D.** a decrease in invasiveness of ∼45%.

Restoration of KIAA1199 expression also altered SW480 cell morphology (Figure 5A). Control SW480 Dest cells, like the parental SW480 line [15], [16], tended to be round, and confluent cultures were characterized by clusters of piled-up cells. In contrast, KIAA1199-V5-expressing cells were flatter with a more epithelial-like aspect. These changes were consistent with the lower proliferation rates (Figure 5B) and reduced cloning efficiency (Figure 5C) observed in KIAA1199-V5 cells. The KIAA1199-expressing cells also displayed decreased *in vitro* invasiveness (Figure 5D), but their directional migration in the in vitro scratch test was not significantly different from that of controls (data not shown). These cellular phenotypes were confirmed in Clone 18, which also expressed KIAA1199 constitutively, but they could not be consistently reproduced by doxycycline-induced KIAA1199 expression in Clone 13.

## Discussion

Using colon cancer cell lines with endogenously or ectopically expressed KIAA1199, we carried out subcellular localization, gene expression, and proteomic studies aimed at delineating the functions of this currently uncharacterized protein. Our findings show that KIAA1199 is a glycosylated protein located predominantly in the perinuclear space (probably the ER, including both the outer nuclear membrane and ER tubules) and plasma membrane. It is also secreted into the extracellular environment (and additional studies are being planned to explore its functions in that milieu). Collectively, these features are suggestive of a protein with functions in distinct membranous cell compartments and they are completely in line with the results of our 2-pronged high-throughput analysis. The lists we compiled of genes encoding putative KIAA1199 interactors and genes whose transcription is significantly up- or downregulated by KIAA1199 expression (Tables S1 and S2) both point to *organelles* and/or *cytoplasm* as KIAA1199’s probable location within the cell (Table 1). Indeed, the 11 genes on both lists all encode proteins known to be localized in these compartments (ACADVL, CANX, EIF3E, EPHA2, FASN, HPRT1, ITPR3, MFGE8, PSMB2, TAGLN2, and TKT–12.4% of the putative interactor proteins).

Both lists also exhibit strong enrichment for components of *cell membrane-*associated pathways (Table 1). The cell-membrane receptor *e*phrin A2 (EPHA2), for example, interacts with KIAA1199, and EPHA2 transcript and protein levels increased slightly upon expression of KIAA1199. The EPHA2 receptor plays key roles in cell adhesion and migration, cytoskeletal organization, mitogenic and survival signaling (e.g., MAP/ERK and PI3K pathways), angiogenesis, and tumor neovascularization [17], and it has been extensively investigated as a therapeutic target in pre-clinical cancer models [18]. EPHA2 and ephrins can produce opposite effects via bidirectional and unconventional signaling modalities, which–to further complicate matters–vary considerably from one cell type to another [19]. This makes it very difficult at this stage of our research to speculate on the outcome of the KIAA1199/EPHA2 interaction in our SW480 clones, in particular, whether it is in any way responsible for the phenotype of KIAA1199-expressing cells shown in Figure 5. Another membrane receptor, the *ITGA6/ITGB4 (alpha-6/beta-4) integrin complex,* also appeared in both our lists. Its abnormal function has been implicated in the progression of carcinomas (Table 1), including those of the colon [20]. When bound by extracellular matrix molecules like laminin, ITGA6/ITGB4 functions with growth factor receptors to bring about hemidesmosomal and cytoskeletal rearrangements and trigger various forms of intracellular signaling (e.g., MAP/ERK and PI3K) that lead to gene expression changes [20]–[22]. A very recent study indicates that KIAA1199 is involved in hyaluronan catabolism in the dermis of the skin and arthritic synovium [23]. Its effects, which are upregulated by histamine and down-regulated by TGFβ1, are mediated by the cell membrane-associated, clathrin-coated pit pathway. Interestingly, combined analysis of our microarray and proteomic data also pointed to the *clathrin-coated vesicle cycle*, *Histamine H1 receptor signaling*, and *TGFβ dependent* mechanisms (Table 1). Finally, PLXNA2, a membrane receptor involved in cell adhesion and motility [24], was found to interact with KIAA1199 in a study based on yeast two-hybrid screens [25]. We cannot confirm or exclude such interaction because on the basis of our microarray screen, PLXNA2 does not appear to be expressed in SW480 cells.

Links between KIAA1199 and branches of the *G-protein signaling* pathway also emerged from our transcriptional and proteomic data sets. The list of putative KIAA1199 interactors was enriched for G proteins that mediate the effects of the S1P (sphingosine-1-phosphate)/S1P receptor (S1PR1, 2 and 3) axes (Table 1). In concert with growth factor receptors, S1PRs regulate tumor cell growth, survival, motility and metastasis, as well as vascular permeability and angiogenesis [26], [27]. Interestingly, signaling through S1PR2 also modulates vascularization of the cochlea, where murine *KIAA1199* mRNA is highly expressed [28]. In fact, *S1pr2*−/− mice are deaf [29], [30], and missense variants in the *KIAA1199* gene are associated with nonsyndromic hearing loss in humans [28].

The lists of GO pathway maps, processes, and molecular functions point strongly toward the ER and functions linked to this organelle, like protein binding, folding, transport, and localization (Table 1). As the major reservoir of intracellular Ca^2+^ in epithelial cells, the ER also plays an essential role in the Ca^2+^ signaling that regulates many cell functions [31]. Inositol 1,4,5-triphosphate receptors (ITPRs), ligand-gated ion channels that are activated by cytosolic Ca^2+^ and IP3, are localized in intracellular membranes, where they mediate the mobilization of intracellular Ca^2+^ stores. ITPR3 was among the putative KIAA1199 interactors we identified and validated, and expression of *ITPR3* mRNA was moderately upregulated upon KIAA1199 expression in microarray experiments. These channels are found on ER membranes of most animal cells, and they are key players in Ca^2+^ signaling [32], [33].

The strong representation of Ca^2+^ signaling in our data sets also points to a potential role of KIAA1199 in the Wnt network (see Table 1). For example, intracellular Ca^2+^ release upon ligand binding is mediated by *trimeric G-proteins*, which are involved in all three components of the Wnt network, i.e., the canonical Wnt/CTNNB1(beta-catenin) pathway, the Wnt/calcium pathway, and the Wnt/Jun N-terminal kinase pathway [34]–[36]. A*ctivation of PKCs via G-protein-coupled receptors* requires diacylglycerol and Ca^2+^, and it plays roles in different Wnt pathways [36]–[40]. Canonical Wnt signaling is inhibited by G protein/Ca^2+^-mediated nuclear export and calpain-mediated degradation of CTNNB1 [41] and by Wnt/CTNNB1-antagonizing NKD proteins [42]–[44]. Accordingly, we found that KIAA1199 expression increased *NKD1* (p  = 0.04) and *CAPN1* (calpain large subunit; p  = 0.03) transcription, and the calpain small subunit (CAPNS1) appears to interact with KIAA1199 (Table S1). These findings are consistent with the results of our GO analysis, which revealed enrichment of *MYC-associated transcription regulation*, although KIAA1199 expression had no significant effect on the expression of MYC mRNA or protein (data not shown). Both lists (Table 1) also displayed enrichment for *Wnt effects on cytoskeletal remodeling* and for *transcriptional regulation by HNF4A*, another key regulator of intestinal epithelium homeostasis that modulates Wnt/CTNNB1 signaling [45].

It is premature to speculate on how KIAA1199’s possible functions in the complex Wnt network might affect canonical Wnt/CTNNB1 signaling in colorectal tissues. However, our data suggest that KIAA1199 is a Wnt signaling target (Figure S2B; see also Ref. [1]) and that its expression can downregulate Wnt signaling via a negative feedback mechanism. CTNNB1-dependent transcriptional activity was significantly (albeit incompletely) inhibited by ectopic expression of KIAA1199 in HEK293 cells, and in SW480 clones nuclear and cytoplasmic levels of CTNNB1 dropped considerably when KIAA1199 expression (untagged as well as V5- or GFP-tagged forms) was restored. These findings are fully consistent with one another despite the markedly different subcellular localizations of CTNNB1 in these two cell lines. In HEK293 cells, CTNNB1 immunostaining typically exhibits a cell-border, CDH1-associated pattern, whereas SW480 cells, which harbor a truncated APC (adenomatous polyposis coli) protein, generally have inordinately high CTNNB1 levels in the cytoplasm and nucleus [46]. Restoration of APC expression in SW480 cells is followed by translocation of CTNNB1 and CDH1 to the cell membrane and diminished Wnt signaling [47]. The drop in cytoplasmic and nuclear CTNNB1 levels we observed in SW480 cells following KIAA1199 expression was not associated with this type of translocation. Furthermore, the restored expression had no detectable impact on MYC mRNA or protein levels in these cells. It remains to be seen whether KIAA1199 modulates CTNNB1 expression patterns in SW480 cells via non-canonical Wnt signaling pathways.

Regardless of its underlying mechanisms, this modulation could have important effects on gene transcription and phenotypic features like cell proliferation and adhesion. Upregulated KIAA1199 expression during colorectal transformation [1] could restrain cell growth by negatively regulating Wnt signaling. Indeed, upregulated *KIAA1199* transcription in human renal cell carcinoma cells [48] and cultured myotubes [49] is associated with cellular or gene-expression features of cell mortality/senescence or apoptosis, and these findings are consistent with the diminished proliferation, cloning efficiency, and invasiveness we observed in SW480 clones that constitutively express KIAA1199. Additional work is needed, however, to clarify the link between the protein’s effects on cell behavior and its impact on Wnt signaling.

Our findings contradict recent findings by Birkenkamp-Demtroder et al. that shRNA-mediated repression of KIAA1199 expression attenuates Wnt signaling in parental SW480 cells [3]. Endogeneous KIAA1199 expression is negligible or absent in molecularly authenticated SW480 cells, as clearly shown by our own data (Figure 1, Figure S1) and also those of Birkenkamp-Demtroder et al. (Supplementary Figure 2a, lanes 2 and 3). It is therefore difficult to see how significant knock-down of KIAA1199 expression can be achieved in this cell line. Unfortunately, the authors have not documented their shRNA-mediated repression by Western blotting. (We used stable shRNA transfection in an attempt to knock down endogenous KIAA1199 expression in LS174T cells, which express high endogenous levels of KIAA1199, but the clones produced were not viable.) When Birkenkamp-Demtroder et al. transfected SW480 cells with V5-His-tagged-KIAA1199, their findings seem much more consistent with our own: their microarray analysis showed that this exogenous expression was associated with significant changes in the expression of genes involved in the ephrin and Wnt signaling pathways. More in-depth comparative analysis of microarray findings from our study and theirs is not currently possible since the raw data from the latter study do not appear to have been deposited in a publicly available database (or there is no information to this effect in their article).

In conclusion, although further work is obviously needed, our data on the restoration of KIAA1199 expression provide useful guidance for future work on this membranous and secreted protein, which appears to be involved in the modulation of ER processes and in Ca^2+^, G-protein, ephrin, and Wnt signaling. Future studies based on other cell models are already being planned to clarify the impact of KIAA1199 on Wnt signaling. Later, the tumorigenicity and metastatic potential of KIAA1199-expressing SW480 cells might be compared with those of non- KIAA1199-expressing control cells in murine xenograft models. Furthermore, because KIAA1199 is a secreted protein, it will be interesting to see if full-length or cleaved forms of the protein can be detected in the blood of the tumor-bearing mice. These experiments could furnish proof of principle for developing KIAA1199 as a secreted biomarker for colorectal neoplasia.

## Supporting Information

Figure S1 **Expression of KIAA1199 mRNA and protein in colorectal tissues and cell lines.** **A.** Expression values for *KIAA1199* mRNA in 32 samples of normal colorectal mucosa, 32 colorectal adenomas, 25 colorectal cancers, and 8 colorectal cancer cell lines (Affymetrix U133Plus2.0 gene expression data from a previous study of ours [1]) **B.** Western blots showing KIAA1199 protein expression in the 8 cell lines. Beta-tubulin (TUBB) was used as loading control.
(TIF)

Figure S2 **KIAA1199 is an** ***N*** **-linked glycoprotein and a putative target of Wnt signaling. A.** Western blot comparing KIAA1199 mobility in whole cell extracts from LS174T cells that were untreated, denatured by boiling at 100°C followed by PNGaseF treatment, or treated with PNGaseF alone. Mobility in the latter two extracts was similarly increased (vs. that observed in the untreated extract), suggesting that *N*-linked sugar residues on KIAA1199 were accessible to the enzyme even without prior denaturation. TUBB was used as loading control. **B.** Western blot: Doxycycline induction of dominant negative TCF4 (TCF4-DN) expression reduces expression of KIAA1199 and MYC proteins in LS174T colon cancer cell clone 8 (LS174T/L8; kindly provided by Dr. Hans Clevers, Hubrecht Institute, Utrecht, The Netherlands). ERCC3 (TFIIH p89) was used as loading control.
(TIF)

Figure S3 **Partial colocalization of KIAA1199 and ITPR3 in the ER.** In general, the anti-KIAA1199 antibodies performed poorly in immunofluorescence experiments. However, a clear perinuclear staining in SW480 KIAA1199-Cl.18 cells strongly pointed to a localization of this protein in the ER. A similar staining pattern was detected with antibodies against ITPR3, which is a well-known ER protein. At this cellular localization, partial overlap of KIAA1199 and ITPR3 staining was detectable in merged images, supporting the IP findings shown in Figure 2D.
(TIF)

Figure S4 **mRNA and protein expression levels of EPHA2 and ITPR3 in SW480 cell clones with or without KIAA1199.** In triplicate microarray experiments, both *EPHA2* and *ITPR3* mRNA levels were found to be upregulated upon expression of KIAA1199 (panel **A**), but in qRT-PCR and Western blotting experiments appreciable increases were seen only in the expression of EPHA2 mRNA (panel **B**) and protein (panel **C**).
(TIF)

Figure S5 (**A**) **Ectopic** **KIAA1199 expression downregulates CTNNB1 expression in HEK293 cells**. Cells were trasfected with an empty vector, Myc-tagged wild-type (CTNNB1^wt^) or Myc-tagged constitutively active beta-catenin (CTNNB1^T41A^), with (lanes 4–6) or without (lanes 1–3) KIAA1199. Western blotting performed 48 h after transfection revealed significant decreases in KIAA1199-transfected cells in the expression of both wild-type CTNNB1 (lane 5 vs. lane 2) and active CTNNB1 (lane 6 vs. lane 3). (**B**) **Ectopic KIAA1199 expression in SW480 colon carcinoma cells produces only moderate reductions in CTNNB1-driven transactivation of TCF-responsive promoter**. Left: SW480 Dest and SW480 KIAA1199 Cl.18 cells were transfected with either TOPflash or FOPflash reporters, and relative luciferase (RLU) activity was assayed 36 hours later. Right: TOP/FOP ratio reflects TCF-specific activation. Data represent the means ± SD of experiments performed in quadruplicate.
(TIF)

Table S1 **List of putative KIAA1199 interactors identified by MS after immunoprecipitation.** Molecular weight, cellular localization, functions, and number of unique peptide hits obtained on MS for each putative interactor is reported.
(PDF)

Table S2 **Genes whose expression significantly changed (p value <0.025, fold change ≥1.2) upon doxycycline-induced expression of KIAA1199 in SW480 Clone 13.**
(PDF)
