# Supplementary figures and images for: Early Insights into the Function of KIAA1199, a Markedly Overexpressed Protein in Human Colorectal Tumors
Source: PLoS One. 2013 Jul 23;8(7):e69473. doi: 10.1371/journal.pone.0069473 (PMC3720655; doi:10.1371/journal.pone.0069473)

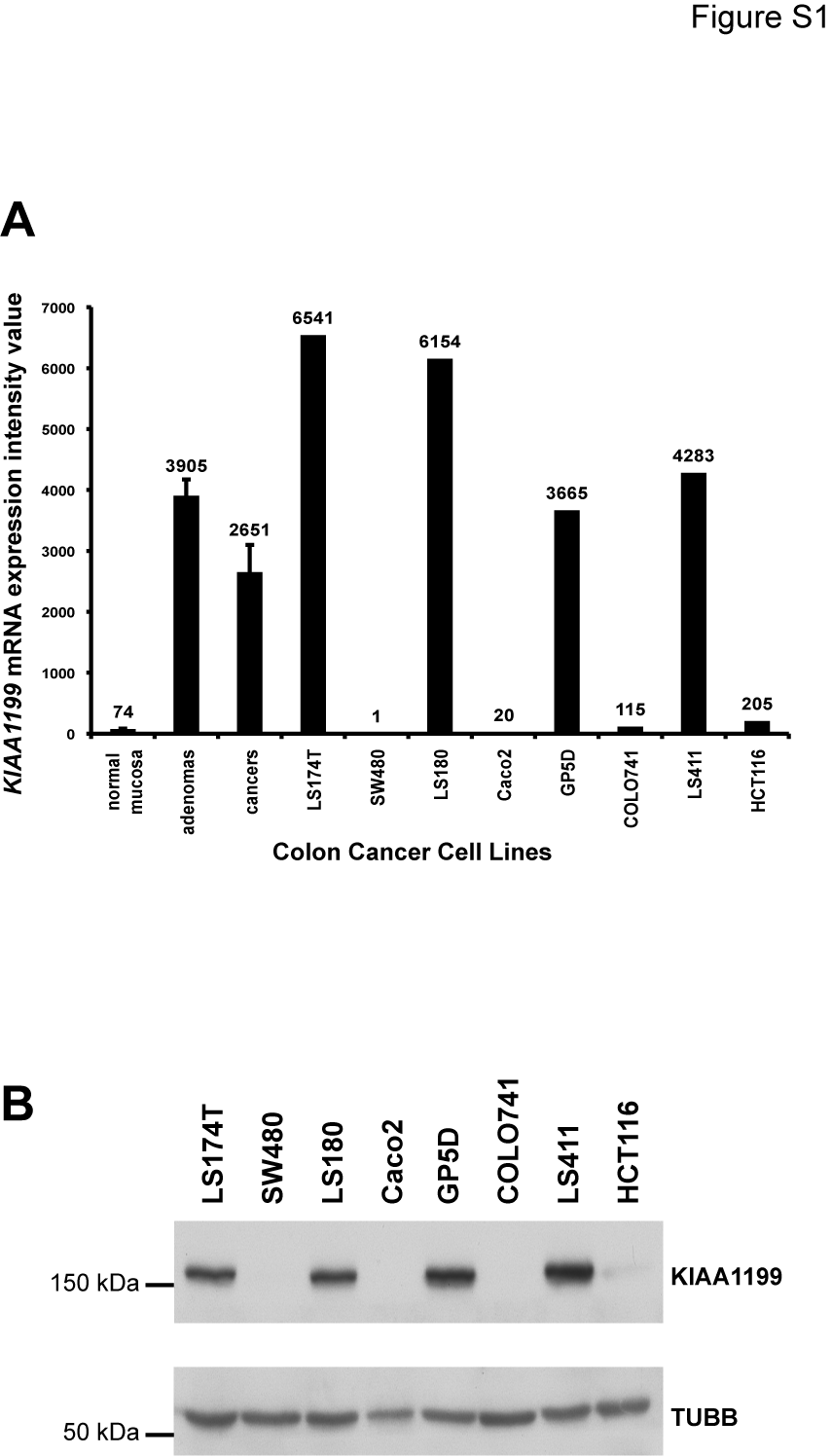

Supplement: Figure S1 — Expression of KIAA1199 mRNA and protein in colorectal tissues and cell lines. A. Expression values for KIAA1199 mRNA in 32 samples of normal colorectal mucosa, 32 colorectal adenomas, 25 colorectal cancers, and 8 colorectal cancer cell lines (Affymetrix U133Plus2.0 gene expression data from a previous study of ours [1]) B. Western blots showing KIAA1199 protein expression in the 8 cell lines. Beta-tubulin (TUBB) was used as loading control. (TIF) [file pone.0069473.s001.tif]

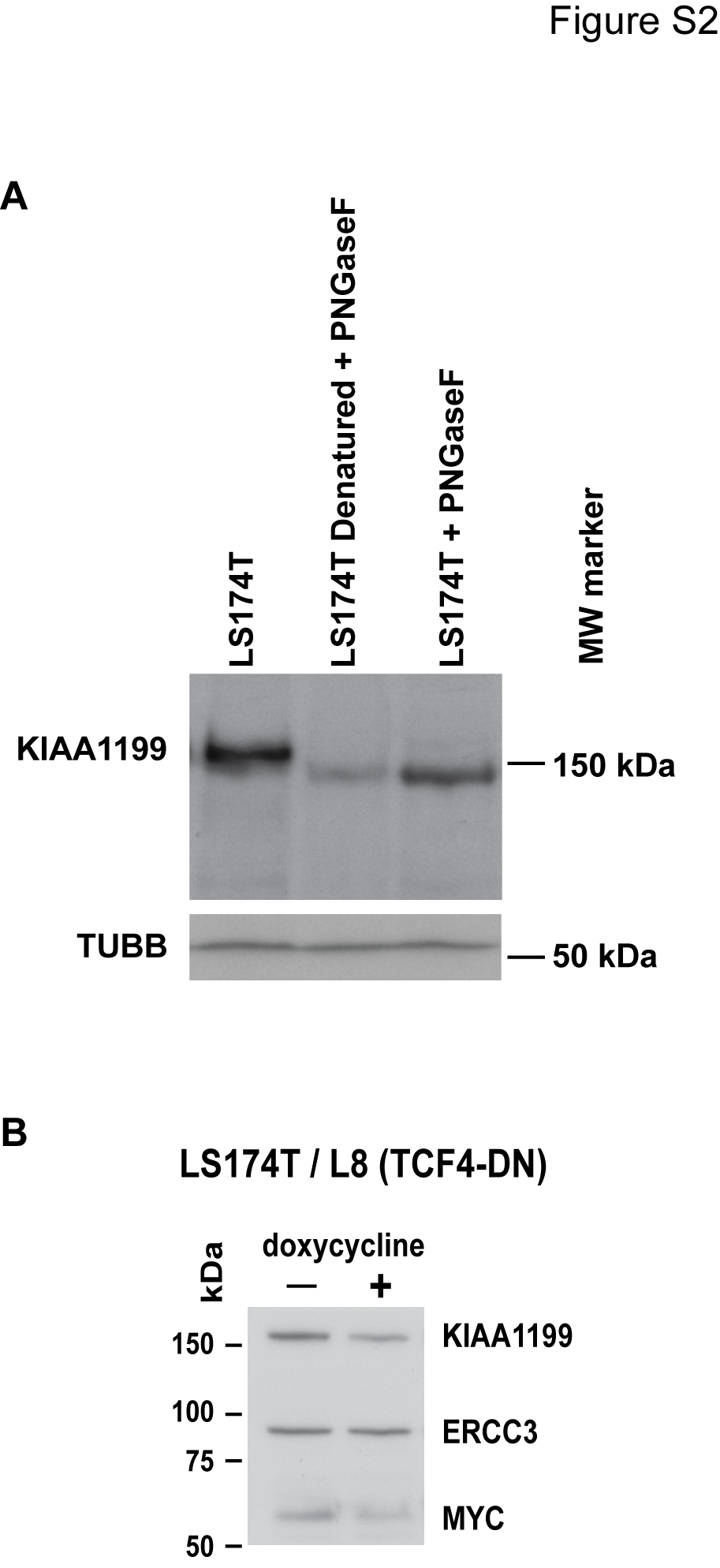

Supplement: Figure S2 — KIAA1199 is an N -linked glycoprotein and a putative target of Wnt signaling. A. Western blot comparing KIAA1199 mobility in whole cell extracts from LS174T cells that were untreated, denatured by boiling at 100°C followed by PNGaseF treatment, or treated with PNGaseF alone. Mobility in the latter two extracts was similarly increased (vs. that observed in the untreated extract), suggesting that N-linked sugar residues on KIAA1199 were accessible to the enzyme even without prior denaturation. TUBB was used as loading control. B. Western blot: Doxycycline induction of dominant negative TCF4 (TCF4-DN) expression reduces expression of KIAA1199 and MYC proteins in LS174T colon cancer cell clone 8 (LS174T/L8; kindly provided by Dr. Hans Clevers, Hubrecht Institute, Utrecht, The Netherlands). ERCC3 (TFIIH p89) was used as loading control. (TIF) [file pone.0069473.s002.tif]

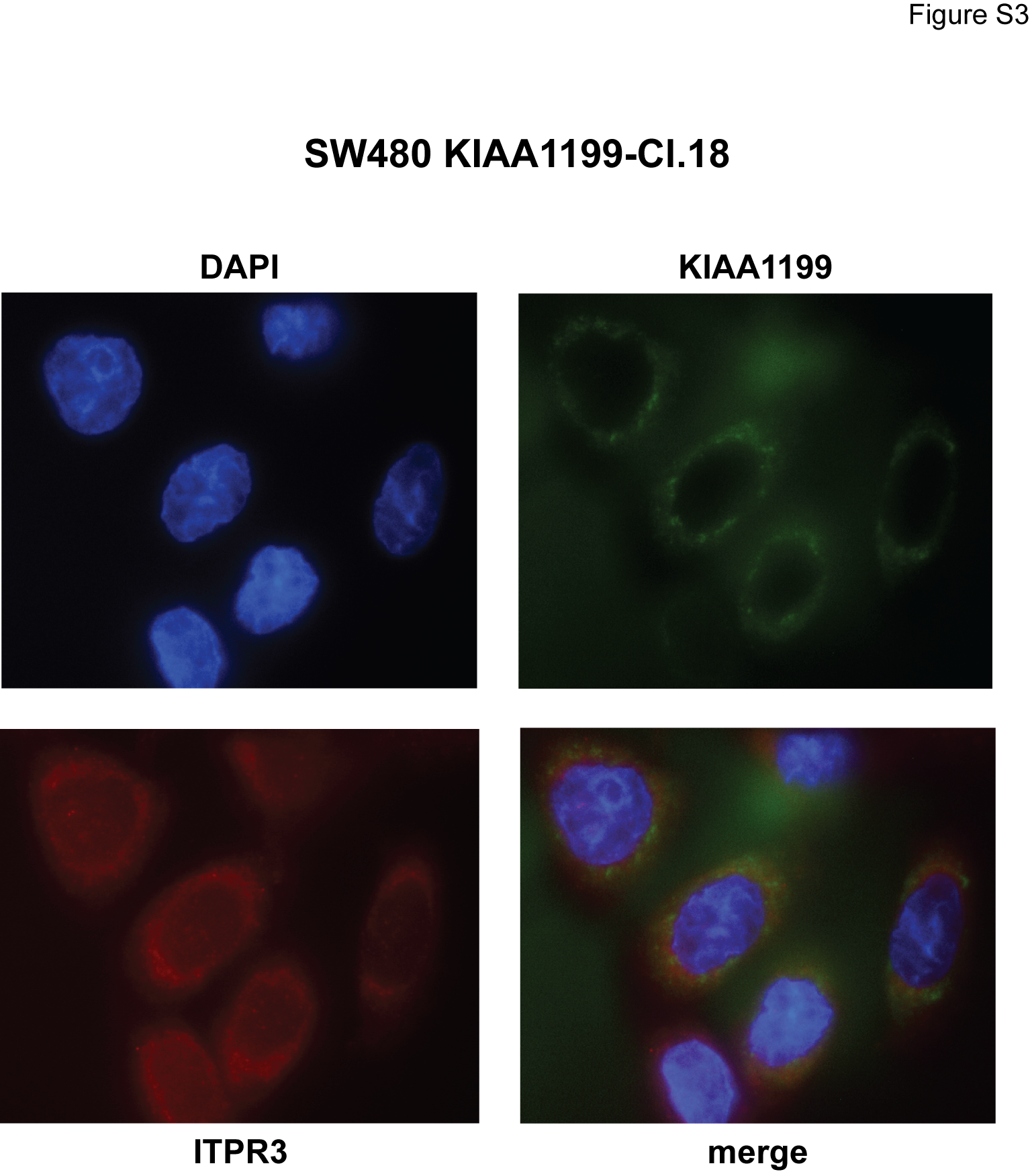

Supplement: Figure S3 — Partial colocalization of KIAA1199 and ITPR3 in the ER. In general, the anti-KIAA1199 antibodies performed poorly in immunofluorescence experiments. However, a clear perinuclear staining in SW480 KIAA1199-Cl.18 cells strongly pointed to a localization of this protein in the ER. A similar staining pattern was detected with antibodies against ITPR3, which is a well-known ER protein. At this cellular localization, partial overlap of KIAA1199 and ITPR3 staining was detectable in merged images, supporting the IP findings shown in Figure 2D. (TIF) [file pone.0069473.s003.tif]

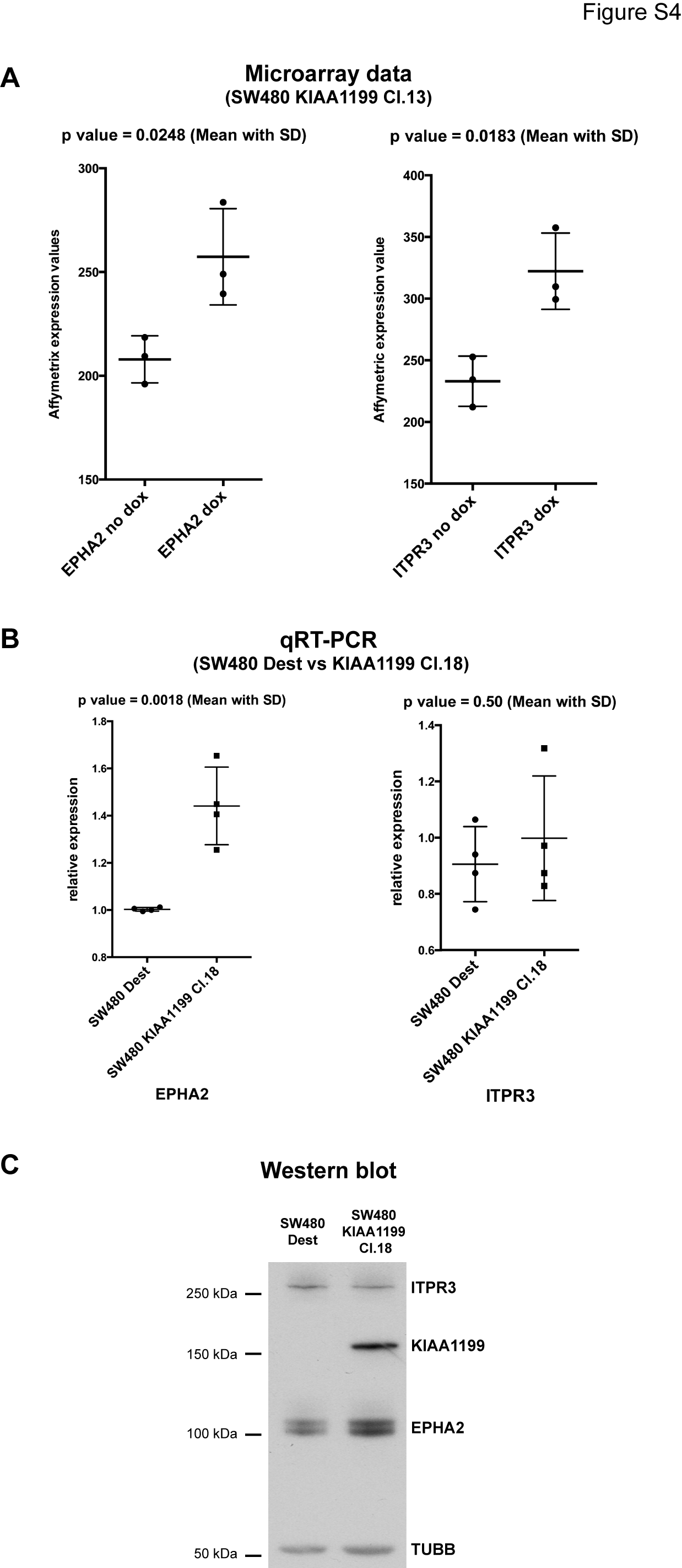

Supplement: Figure S4 — mRNA and protein expression levels of EPHA2 and ITPR3 in SW480 cell clones with or without KIAA1199. In triplicate microarray experiments, both EPHA2 and ITPR3 mRNA levels were found to be upregulated upon expression of KIAA1199 (panel A), but in qRT-PCR and Western blotting experiments appreciable increases were seen only in the expression of EPHA2 mRNA (panel B) and protein (panel C). (TIF) [file pone.0069473.s004.tif]

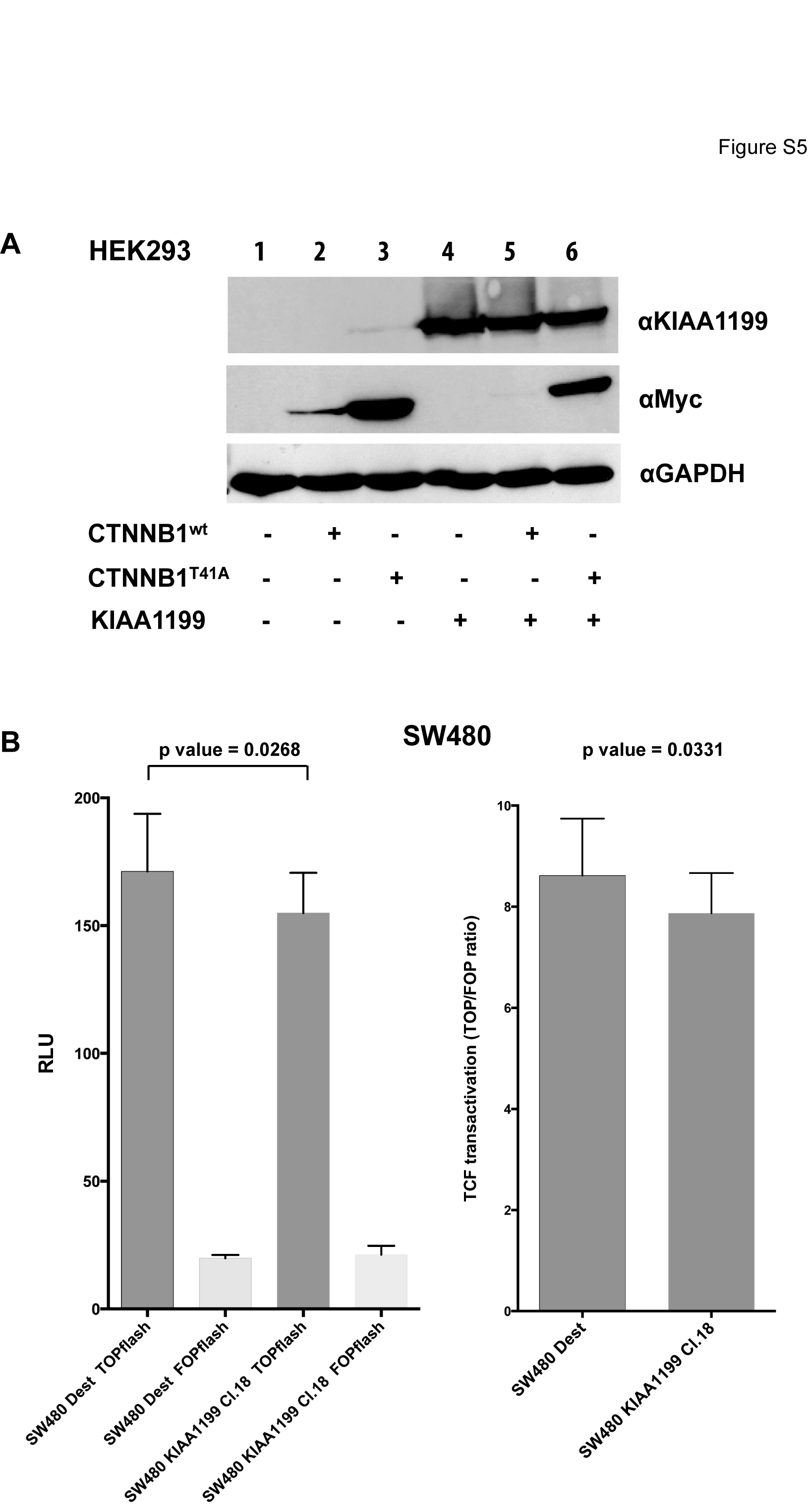

Supplement: Figure S5 — (A) Ectopic KIAA1199 expression downregulates CTNNB1 expression in HEK293 cells. Cells were trasfected with an empty vector, Myc-tagged wild-type (CTNNB1wt) or Myc-tagged constitutively active beta-catenin (CTNNB1T41A), with (lanes 4–6) or without (lanes 1–3) KIAA1199. Western blotting performed 48 h after transfection revealed significant decreases in KIAA1199-transfected cells in the expression of both wild-type CTNNB1 (lane 5 vs. lane 2) and active CTNNB1 (lane 6 vs. lane 3). (B) Ectopic KIAA1199 expression in SW480 colon carcinoma cells produces only moderate reductions in CTNNB1-driven transactivation of TCF-responsive promoter. Left: SW480 Dest and SW480 KIAA1199 Cl.18 cells were transfected with either TOPflash or FOPflash reporters, and relative luciferase (RLU) activity was assayed 36 hours later. Right: TOP/FOP ratio reflects TCF-specific activation. Data represent the means ± SD of experiments performed in quadruplicate. (TIF) [file pone.0069473.s005.tif]
