# Supplementary material for: Early Insights into the Function of KIAA1199, a Markedly Overexpressed Protein in Human Colorectal Tumors
Source: PLoS One. 2013 Jul 23;8(7):e69473. doi: 10.1371/journal.pone.0069473 (PMC3720655; doi:10.1371/journal.pone.0069473)
Supplement: Table S1 — List of putative KIAA1199 interactors identified by MS after immunoprecipitation. Molecular weight, cellular localization, functions, and number of unique peptide hits obtained on MS for each putative interactor is reported. (PDF) [file pone.0069473.s006.pdf]

Table S1. List of putative KIAA1199 interactors identified by MS after immunoprecipitation. Molecular weight, cellular localization, functions, and number of unique peptide hits obtained on MS for each putative interactor is reported.

Protein symbol *	Mol. wt.	Localization	Pathway	IP/MS experiment number †					
				1		2		3	
				Control	IP	Control	IP	Control	IP
**FAT1**	506 kDa	Membrane, Cell junctions	Calcium ion binding, cell adhesion, homophilic cell adhesion, cell-cell signaling, anatomical structure morphogenesis	0	1	0	13	0	2
**ITPR3**	304 kDa	Intracellular Membranes, ER, nucleus, nucleoplasm,	Calcium channel activity, inositol hexakisphosphate binding, G-protein coupled receptor protein signaling pathway	0	0	0	1	0	1
**SERPINE1**	45 kDa	Plasma Membrane, Extracellular Matrix, Soluble fraction	Response to ROS, Chronological cell aging, platelet degranulation, blood coagulation, regulation of receptor activity	0	6	1	13	1	6
**GART**	108 kDa	Cytoplasm	Purine metabolism, 'de novo' IMP biosynthetic process	0	1	0	2	0	2
**UBR5**	308 kDa	Nucleus, intracellular, Soluble fraction	Cell proliferation, progesterone receptor signaling pathway, response to DNA damage stimulus, ubiquitin-dependent protein catabolic process, protein modification process	0	0	0	10	0	2
**HSP90B1**	92 kDa	ER, Microsome, Cytosol	Response to hypoxia or stress, protein folding, anti-apoptosis, protein transport	0	10	0	8	1	4

**GNAI2**	40 kDa	Plasma Membrane, Nucleus, cytosol	Activation of MAPKK activity, adenosine receptor signaling pathway, G-protein coupled receptor protein signaling pathway, inhibition of adenylate cyclase activity by G-protein signaling pathway	0	0	1	19	0	8
**VCP**	89 kDa	Proteasome complex, ER	Receptor binding, ubiquitin-dependent protein catabolic process, double strand break repair	0	7	1	11	3	5
**NME2**	17 kDa	Nucleus, Cytoplasm, lamellipodium	Purine metabolism, Pyrimidine metabolism, regulation of transcription, DNA-dependent; cell adhesion	0	2	1	4	0	5
**GNAI3**	41 kDa	Intracellular, Plasma Membrane, Cytoplasm, Golgi apparatus	G-protein activator peptide, Inhibits Gs & stimulates Gi activity, Selective, competitive P2X antagonist	0	0	0	7	0	1
**ANXA6**	76 kDa	Cytoplasm, Apical Plasma Membrane, Melanosome, Perinuclear region of cytoplasm	Calcium Ion Binding, Calcium dependent phospholipid binding, calcium ion transport, regulation of muscle contraction	0	11	0	2	0	0
**LGALS3**	26 kDa	Extracellular matrix, Nucleus, cytoplasm, Mitochondrial Inner Membrane, plasma Membrane	Skeletal system development, Cell differentiation, Extracellular Matrix Organization.	0	2	0	6	1	4

**STOML2**	39 kDa	Cytoplasm, Mitochondrion, Mitochondrial Inner Membrane, Cytoskeleton, Membrane	Receptor- binding	0	2	0	6	1	4
**CANX**	68 kDa	Cytoplasm, ER (Lumen, Membrane), RER	Calcium Ion Binding, Protein folding, ageing, post translational protein modification	1	8	1	7	1	5
**MDH2**	36 kDa	Nucleus, mitochondrial inner membrane, mitochondrial matrix	Protein binding, malate dehydrogenase activity, oxidoreductase activity	0	8	0	6	2	2
**IPO5**	124 kDa	Nucleus, Nuclear pore, nucleolus, cytoplasm	NLS-bearing substrate import into nucleus, intracellular protein transport,interspecies interaction between organisms	0	2	1	7	1	7
**ITGB4**	202 kDa	Plasma Membrane, Integrin Complex	Cell communication, cell adhesion, cell-matrix adhesion, integrin-mediated signaling pathway, multicellular organismal development	0	2	2	5	0	6
**SLC25A3**	40 kDa	Mitochondrial inner membrane, integral to plasma membrane	symporter activity, phosphate carrier activity	1	5	3	20	9	6

**GPI**	63 kDa	Extracellular space, nucleus.	Angiogenesis, Carbohydrate Metabolic process, growth factor activity, isomerase activity	0	4	0	7	3	4
**LRP1**	505 kDa	Plasma Membrane, Nucleus, Cytoplasm, Endosome	Receptor-mediated endocytosis, Cell Proliferation, Aging, Multicellular Organismal Development,activation of protein kinase C activity by G-protein coupled receptor protein signaling pathway	0	0	0	2	0	2
**ATP6V1A**	68 kDa	Mitochondrion, cytosol, microvillus, integral to plasma membrane	hydrolase activity, acting on acid anhydrides, catalyzing transmembrane movement of substance	0	2	0	1	0	1
**P4HB**	57 kDa	ER-Golgi intermediate compartment, ER lumen	Protein disulfide isomerase activity, procollagen-proline 4-dioxygenase activity	0	4	1	9	3	5
**ATIC**	65 kDa	Mitochondrion, Cytosol	Purine metabolism/biosynthesis	0	0	0	2	0	5
**TPI**	27 kDa	Soluble fraction, Nucleus, Cytosol	Glycolysis, Gluconeogenesis, Pentose-Phosphate Shunt	1	7	1	4	7	5
**DNAJC7**	56 kDa	Nucleus, Cytoplasm, Cytoskeleton	Chaperone cofactor-dependent protein refolding	0	0	0	3	0	2
**HYOU1**	111 kDa	ER, ER Lumen	Response to hypoxia/stress, ATP binding	0	2	0	1	1	3

**PSMD1**	106 kDa	Proteasome complex, Proteasome regulatory particle	Cell cycle check-point, G1/S transition, S-phase, M/G1 transition of mitotic cycle	0	1	0	3	1	3
**ITGA6**	127 kDa	Plasma membrane, cell-cell adherens junction, integrin complex,	Cell-substrate junction assembly, integrin-mediated signaling pathway, Blood coagulation, cell-adhesion, cell-matrix adhesion	0	2	0	6	2	4
**HSPA4**	70 kDa	Nucleus, Cytoplasm	Response to unfolded protein, response to heat, cellular chaperone-mediated protein complex assembly, protein import into mitochondrial outer membrane, mitochondrial outer membrane translocase complex assembly	0	3	1	3	2	8
**AP1G1**	91 kDa	Golgi membrane, cytoplasm, lysosomal membrane, trans-golgi network	Intracellular protein transport, endocytosis, viral reproduction, post-Goldi vesicle mediated transport	0	1	0	1	0	3
**EPHA2**	108 kDa	Plasma Membrane, Integral to plasma Membrane	Skeletal system development, Blood vessel development, vasculogenesis, protein phosphorylation, apoptosis, ephrin receptor signaling pathway	0	0	0	2	0	2
**PPP6R3**	98 kDa	Nucleus, Cytoplam	Regulation of phosphoprotein phosphatase activity	0	1	5	11	2	10

**TKT**	68 kDa	Nucleus, Cytoplam	Glucose, thiamin metabolic process	0	1	0	1	0	2
**PSMD11**	47 kDa	Proteasome complex	Protein binding	0	0	1	8	0	2
**GARS**	83 kDa	Soluble fraction cytoplasm, mitochondrion	Translation, tRNA aminoacylation for protein translation, glycyl-tRNA aminoacylation, cell death, gene expression	0	2	1	3	1	4
**TARDBP**	45 kDa	Nucleus	Transcription from RNA polymerase II promoter, mRNA processing, cell death, RNA splicing, negative regulation by host of viral transcription	0	1	1	5	1	2
**GSTO1**	28 kDa	Cytoplasm, cytosol	Monodehydroascorbate reductase (NADH) activity, glutathione transferase activity	0	1	0	1	0	1
**BSG**	42 kDa	Golgi membrane, Mitochondrion, Plasma Membrane, Sarcolemma	Pyruvate metabolic process, cell surface receptor linked signaling pathway, embryo implantation, blood coagulation, odontogenesis of dentine-containing tooth	0	2	2	6	0	1
**IPO8**	120 kDa	Nucleus, Cytoplasm	Intracellular protein transport, signal transduction, Ran GTPase binding	0	0	0	1	0	2
**CTSD**	45 kDa	Extracellular matrix, mitochondrion, lysosome,	Autophagic vacuole assembly, proteolysis, cell death	0	2	0	3	3	1
**RAB5C**	23 kDa	Intracellular, endosome, plasma membrane	GTP catabolic process, intracellular protein transport, nucleocytoplasmic transport, endosome organization, signal transduction	0	1	0	3	2	1
**PGK1**	45 kDa	Cytoplasm	Carbohydrate metabolic process, ATP binding	0	7	0	4	3	3

**TIMM50**	40 kDa	Nucleus, mitochondrion, mitochondrial inner membrane, mitochondrial inner membrane presequence translocase complex , membrane	Release of cytochrome c from mitochondria, protein dephosphorylation, mitochondrial membrane organization, protein transport, transmembrane transport	1	5	8	9	1	5
**EZR**	69 kDa	Ruffle, uropod, nucleolus, cytoplasm, cytosol	Cytoskeletal anchoring at plasma membrane, leukocyte cell-cell adhesion, axon guidance, regulation of cell shape, membrane to membrane docking	1	8	0	6	5	3
**RBP1**	16 kDa	Cytoplasm	Vitamin A metabolic process, transport, regulation of granulocyte differentiation, response to vitamin A, retinol metabolic process	1	6	0	3	0	0
**CCT2**	57 kDa	Nucleus but NOT nucleolus, cytoplasm	Protein folding, cellular protein metabolic process, 'de novo' posttranslational protein folding	0	4	0	7	7	4
**MSN**	68 kDa	Nucleus, nucleolus, cytoplasm	Structural constituent of cytoskeleton, actin binding	0	8	0	2	0	0
**GPC1**	62 kDa	Extracellular matrix, plasma membrane	Axon guidance	0	0	0	4	0	1
**CCT8**	60 kDa	Cytoplasm, microtubule org. center	ATP binding, ATPase activity, unfolded protein binding	0	5	2	9	9	10

**RARS**	75 kDa	Nucleus but NOT nucleolus, cytoplasm, mitochondrion	Arginine-tRNA ligase activity, nucleotide binding, protein binding	0	4	5	6	0	6
**SNAP23**	23 kDa	Nucleus but NOT nucleolus, microsome, Golgi apparatus, plasma membrane enriched fraction	Protein binding	0	0	0	8	0	1
**SMC4**	147 kDa	Nucleus but NOT nucleolus, condensin complex	Nucleotide binding, nucleoside-triphosphatase activity, protein binding, protein heterodimerization activity	0	3	1	5	5	4
**CD109**	162 kDa	Plasma membrane, anchored to membrane, extracellular region	Serine-type endopeptidase inhibitor activity	0	0	0	7	0	1
**PDIA6**	48 kDa	ER-Golgi intermediate compartment, plasma membrane, melanosome	Protein disulfide isomerase activity, protein binding	0	5	2	8	5	4

**TAGLN2**	22 kDa	Plasma membrane, nuclear membrane	Protein binding	0	2	0	4	7	4
**Septin9**	65 kDa	Cytoplasm, cytoskeleton	GTP catabolic process, protein heterooligomerization, cell cycle, cell division	0	4	1	0	0	2
**PSMA3**	28 kDa	Nucleus, cytoplam, proteasome core complex	Threonine-type endopeptidase activity	0	3	0	1	0	0
**ANXA5**	36 kDa	Cytoplasm, plasma membrane	Phospholipase inhibitor activity, calcium-dependent phospholipid binding	0	1	0	2	2	0
**GNB2**	37 kDa	Plasma membrane, perinuclear region of cytoplasm	G-protein-coupled receptor binding, GTPase activity	0	0	0	5	0	1
**KHSRP**	73 kDa	Nucleus, cytoplasm	DNA binding, RNA binding, protein binding	1	5	0	1	0	0
**MFGE8**	43 kDa	Extracellular space, external side of plasma membrane	Phosphatidylserine binding, integrin binding, calcium ion binding, phosphatidylethanolamine binding	0	0	0	6	0	1
**SMARCC2**	133 kDa	nucleoplasm, chromatin	DNA binding, protein binding, transcription co-activator activity	0	2	0	1	3	4
**CS**	52 kDa	Mitochondrial matrix	Citrate (Si)-synthase activity	0	2	0	2	4	9
**SET**	33 kDa	Nucleoplasm, cytoplasm, ER	Histone binding, transcription repressor activity, protein phosphatase type 2A regulator activity	0	3	0	2	0	0

**HPRT1**	25 kDa	Cytoplasm, cytosol	Hypoxanthine phosphoribosyltransferase activity	0	2	0	2	2	1
**EIF3E**	52 kDa	Nucleoplasm, cytoplasm	Contributes to translation intiation factor activity	0	0	0	4	0	2
**CBR1**	30 kDa	Cytoplasm	Prostaglandin-E2 9-reductase activity, 15-hydroxyprostaglandin dehydrogenase (NADP+) activity	0	1	0	2	0	0
**PSMB2**	23 kDa	Nucleus, cytoplasm, proteasome core complex	Threonine-type endopeptidase activity	0	0	0	2	0	1
**TNPO1**	102 kDa	Nucleus, cytoplasm	Protein transporter activity	0	0	0	2	0	1
**UBE3C**	124 kDa	Nucleus, proteasome complex	Ubiquitin-protein ligase activity	0	0	0	5	0	1
**UGGT1**	177 kDa	ER-Golgi intermediate compartment	UDP-glucose:glycoprotein glucosyltransferase activity	0	1	0	4	0	0
**TLN1**	270 kDa	Cytoplasm, cytosol, centrosome	Actin binding, insulin receptor binding, strucutural constituent of cytoskeleton	0	1	0	1	0	0
**QARS**	88 kDa	Cytoplasm, cytosol, mitochondrial matrix	Glutamine-tRNA ligase activity	0	0	0	4	1	4
**ALDOA**	39 kDa	Actin cytoskeleton, platelet alpha granule lumen	Fructose-bisphosphate aldolase activity, actin binding, tubulin binding	0	1	0	6	4	6
**GANAB**	107 kDa	ER lumen, golgi app., melanosome	Glucan-1,3-alpha-glucosidase activity	0	2	0	9	1	1

**GMPS**	77 kDa	Cytoplasm, cytosol	GMP synthase (glutamine-hydrolyzing) activity, ATP binding	0	3	0	1	1	2
**PRPF6**	107 kDa	Nuceloplasm, spliceosomal complex	Assembly of spliceosomal tri-snRNP, spliceosome assembly, nuclear mRNA splicing, via spliceosome	0	0	1	8	0	1
**ACADVL**	70 kDa	Mitochondrial inner membrane, mitochondrial matrix, mitochondrial nucleoid	Long-chain-acyl-CoA dehydrogenase activity	0	1	2	6	1	8
**UQCRC2**	48 kDa	Mitochondrial respiratory chain complex III	Metalloendopeptidase activity	0	1	2	4	1	4
**OPA1**	112 kDa	Mitochondrion	Nucleotide binding, magnesium ion binding, GTPase activity	0	0	2	8	0	2
**SRRT**	101 kDa	Nucleus, nucleoplasm, cytoplasm	Nucleic acid binding, protein binding	0	0	1	5	0	3
**FASN**	273 kDa	Cytoplasm, mitochondrion, Golgi apparatus, plasma membrane, cytosol	Fatty acid synthase activity	0	0	1	7	0	4
**CAPNS1**	28 kDa	Cytoplasm, plasma membrane	Calcium-dependent cysteine-type endopeptidase activity, protein heterodimerization activity	0	2	1	4	1	1

**DDX39B**	49 kDa	Nucleus, spliceosomal complex	ATP-dependent RNA helicase activity	0	2	0	3	1	1
**POLR2B**	134 kDa	Nucleus, nucleoplasm, DNA-directed RNA polymerase II, core complex	DNA-directed RNA polymerase activity, contributes to protein kinase activity	0	0	0	5	1	4
**CTTND1**	108 kDa	Nucleus, Cytoplasm, Adherens junction	Morphogenesis of a branching structure, Cell adhesion, signal transduction, multicellular organismal development	0	0	0	1	0	1
**PA2G4**	44 kDa	Nucleus but not nucleoplasm, ribonucleoprot ein complex	Ubiquitin protein ligase binding, sequence-specific DNA binding transcription factor activity, RNA binding	0	3	0	3	4	1
**IARS**	145 kDa	Nucleus but not nucleolous, cytoplasm	Isoleucine-tRNA ligase activity, ATP binding	0	4	1	5	4	4
**ACOT9**	50 kDa	Mitochondrion	Acetyl-CoA hydrolase activity	0	0	0	1	0	1

*** All peptides met the following criteria in at least 2 out of 3 experiments: a) presence in the KIAA1199 immunoprecipitates (i.e., IP) and absent in control; or b) present at least four-times more frequently in the KIAA1199 immunoprepitates than in controls.**

† Number of unique peptide hits obtained on MS.
