# Supplementary material for: Early Insights into the Function of KIAA1199, a Markedly Overexpressed Protein in Human Colorectal Tumors
Source: PLoS One. 2013 Jul 23;8(7):e69473. doi: 10.1371/journal.pone.0069473 (PMC3720655; doi:10.1371/journal.pone.0069473)
Supplement: Table S2 — Genes whose expression significantly changed (p value <0.025, fold change ≥1.2) upon doxycycline-induced expression of KIAA1199 in SW480 Clone 13. (PDF) [file pone.0069473.s007.pdf]

Table S2. Genes whose expression significantly changed (p value <0.025, fold change ≥ 1.2) upon doxycycline-induced expression of KIAA1199 in SW480 Clone 13.

Transcript ID	Gene Symbol	RefSeq	p-value (doxycycline vs. control)	Fold change (doxycycline vs. control)
2382970	**EPHX1**	NM_000120	6.10E-05	1.25658
3809732	**NARS**	NM_004539.3	0.000139085	-1.69503
3631517	**THAP10**	NM_020147	0.000292807	-1.25279
3733911	**SSTR2**	NM_001050	0.000312411	-1.33443
3740610	**PRPF8**	NM_006445.3	0.000351469	-1.46223
3468225	**CCDC53**	NM_016053	0.000368365	-1.6111
3432641	**C12orf52**	BC022092	0.000396522	1.2044
3860824	**ZNF569**	NM_152484	0.000409502	-1.29266
2837970	**ADRA1B**	NM_000679	0.000454613	1.26301
2669888	**GORASP1**	NM_031899	0.000644523	1.23781
3316234	**NS3BP**	AF435951	0.000698923	1.42178
2621275	**KLHL18**	NM_025010	0.000709853	1.32045
3604147	**KIAA1199**	NM_018689	0.0008327	3.47319
3821377	**ZNF441**	NM_152355	0.000841967	-1.31725
3235932	**PRPF18**	NM_003675	0.000857099	-1.33081
3015603	**AGFG2**	NM_006076	0.000875494	1.21069
3819599	**HNRNPM**	NM_005968.4	0.000972584	1.67353
2474706	**SLC4A1AP**	NM_018158	0.0010313	1.29866
2831968	**IK**	NM_006083.3	0.00106488	-2.33111
3822122	**NFIX**	NM_002501	0.00108186	1.23673
3831698	**ZNF420**	NM_144689	0.00111942	-1.36974
3630184	**RPL4**	NM_000968.3	0.00112372	-1.25866
3646226	**UNQ3118**	AY358225	0.00116969	-1.26107
3319327	**E1F3F**	NM_003754.2	0.0012004	-1.78875
3203665	**PTENP1**	NR_023917	0.00128995	-1.49164
3250019	**DDX50**	NM_024045	0.0013249	-1.37906
3070499	**RNF133**	NM_139175	0.0013572	1.29476
2328273	**SERINC2**	NM_178865	0.00144492	1.28271
3869361	**ZNF615**	NM_198480	0.00149332	-1.26857
3654699	**NUPR1**	NM_001042483	0.00149652	-1.25034
3275590	**GDI2**	NM_001115156.1	0.00156722	-1.57645
3432333	**PTPN11**	NM_002834	0.00161639	-1.2001
3713575	**PRPSAP2**	NM_002767	0.00166651	-1.30394
3897505	**JAG1**	NM_000214	0.00179587	1.21656
3548772	**TRIP11**	NM_004239	0.00185087	-1.43375
3096448	**FNTA**	NM_002027.2	0.00192917	-1.34012
3214749	**NOL8**	NR_024020	0.00195771	-1.20174
2908762	**RUNX2**	NM_001024630	0.00202544	1.27096

3292448	**HERC4**	NM_022079	0.00203528	-1.23367
2841322	**ATP6V0E1**	NM_003945.3	0.00205389	-2.27778
3176689	**FLJ44082**	NM_207416	0.00207454	-1.31447
2638676	**EAF2**	NM_018456	0.00207909	-1.30072
3165780	**IFT74**	NM_025103	0.00222291	-1.5621
3969081	**TLR7**	NM_016562	0.00227297	-1.40695
3453732	**TUBA1B**	NM_006082	0.00229895	-1.99173
3748126	**ATPAF2**	NM_145691	0.00241491	1.23313
3667702	**UNQ6484**	AY358233	0.00243557	-1.55148
3829964	**ZNF30**	NR_024018	0.00246349	-1.52545
3475511	**DIABLO**	NM_019887	0.00246654	-1.23721
2409507	**SLC6A9**	NM_201649	0.00246822	1.23766
3188147	**OR1J2**	NM_054107	0.00249632	1.35447
3475166	**ANAPC5**	NM_001137559.1	0.00251568	1.35099
3119945	**GRINA**	NM_000837	0.00261111	1.23679
2333671	**ATP6V0B**	NM_001039457.1	0.00268712	1.61
2686776	**RPL24**	NM_000986.3	0.00272529	-1.42416
2360989	**MSTO1**	NM_018116	0.00277497	1.78437
2539765	**ITGB1BP1**	NM_004763	0.00281175	-1.26933
2599911	**C2orf24**	NM_015680.4	0.00282992	1.55705
3363979	**PSMA1**	NM_148976	0.00283523	-1.32954
3334847	**C11orf2**	NM_013265	0.00288648	1.21345
3986672	**ATG4A**	NM_052936	0.00288684	-1.24712
3793588	**C18orf55**	NM_014177	0.00289012	-1.31215
3015553	**MEPCE**	NM_019606	0.00290032	1.25601
2406395	**PSMB2**	NM_001199779.1	0.00293312	-1.3917
2333794	**DMAP1**	NM_019100	0.00293544	1.22155
3059258	**PCLO**	NM_033026	0.00294062	1.22694
3015706	**MOSPD3**	NM_001040097	0.00294068	1.24219
3973556	**CXorf59**	NM_173695.2	0.00295738	-1.24505
3070658	**NDUFA5**	NM_005000	0.00300053	-1.75197
2628482	**FAM19A1**	NM_213609	0.00300571	-1.30627
3057650	**YWHAG**	NM_012479	0.00303374	1.31252
2449922	**ATP6V1G3**	NM_133326	0.00320127	-1.25843
2320472	**CLCN6**	NM_001286	0.00321022	1.26375
3448428	**C12orf11**	NM_018164	0.00324277	-1.27754
2974671	**C6orf192**	NM_052831	0.00326021	-1.51411
2775500	**HNRNPD**	NM_001003810.1	0.00330473	1.35005
3335795	**SART1**	NM_005146.4	0.00338042	1.28322
3333086	**RPLP0**	NM_001002	0.00341255	-1.22835
2438207	**MEF2D**	NM_005920	0.00343095	1.32392
3822805	**TECR**	NM_138501	0.0036021	1.25726
3142485	**IMPA1**	NM_001144878	0.0036576	-1.27872
3577443	**ASB2**	NM_016150	0.00374178	-1.22637
3355860	**KCNJ5**	NM_000890	0.00374776	1.24319
3381682	**CHCHD8**	NM_016565	0.00375057	-1.42524

3347615	**ACAT1**	NM_000019	0.00385855	-1.26418
4019967	**C1GALT1C1**	NM_152692	0.00386131	-1.40809
3817933	**ZNRF4**	NM_181710	0.00386807	-1.22761
3079722	**CRYGN**	NM_144727	0.0038706	1.28026
2775909	**PLAC8**	NM_016619	0.00395353	-1.39008
3537884	**ARID4A**	NM_002892	0.00400383	-1.37399
2600237	**OBSL1**	NM_015311	0.00400511	1.31829
3407229	**AEBP2**	NM_153207	0.0040423	-1.38909
3774635	**FASN**	NM_004104	0.0040471	1.21742
3376046	**LRRN4CL**	NM_203422	0.00411146	-1.26159
2412799	**ORC1L**	NM_004153	0.00425054	1.21624
3831774	**ZNF383**	NM_152604	0.00434804	-1.25707
3629350	**SPG21**	NM_001127889	0.00440238	-1.24942
2383762	**ARF1**	NM_001024226.1	0.00441317	1.20932
3598959	**SMAD3**	NM_005902	0.00447211	1.22007
3910347	**SUMO1P1**	NR_002189	0.00461002	-1.24175
3878934	**NAT5**	NM_016100	0.0046792	-1.27818
3178583	**CKS2**	NM_001827	0.00473185	-1.29848
3464000	**CCDC59**	NM_014167	0.00491631	-1.64207
2950629	**TAPBP**	NM_172208	0.00499274	1.23341
3204903	**SPAG8**	NM_172312	0.00501365	1.35196
3759540	**DCAKD**	NM_024819	0.00508101	1.29729
2901692	**ABCF1**	NM_001025091.1	0.00516348	-1.54492
3304475	**ARL3**	NM_004311	0.00521045	-1.32901
3416492	**HNRNPA1L2**	NM_001011724.1	0.00531014	1.59127
3703598	**FBXO31**	NM_024735	0.0053488	1.21189
3748659	**GRAP**	NM_006613	0.00541432	-2.16327
3226303	**NAIF1**	NM_197956	0.00548953	1.29444
3219621	**CTNNAL1**	NM_003798	0.00558922	-1.25157
2334476	**MAST2**	NM_015112	0.00563783	1.22695
3974556	**ATP6AP2**	NM_005765	0.00565857	-1.27802
3184218	**C9orf6**	NM_017832	0.00566372	-1.38884
3238761	**MSRB2**	NM_012228	0.00570383	-1.21204
3733275	**KCNJ2**	NM_000891	0.00581487	-1.25069
3505449	**MIPEP**	NM_005932	0.00583454	-1.202
3367183	**LIN7C**	NM_018362	0.00584987	-1.42987
3743167	**MED31**	NM_016060	0.00587165	-1.47241
3166844	**CHMP5**	NM_016410	0.00593019	-1.40553
3389529	**KIAA1826**	NM_032424	0.00598323	-1.324
2413203	**LRP8**	NM_004631	0.00599536	1.21248
3323413	**HTATIP2**	NM_001098522	0.00601293	-1.22497
3727510	**STXBP4**	NM_178509	0.00607881	-1.32806
3489350	**CDADC1**	NM_030911	0.00613492	-1.28471
3532353	**FAM177A1**	NM_001079519	0.00615525	-1.39486
3741352	**OR3A2**	NM_002551	0.00616149	-1.30315
2921022	**GPR6**	NM_005284	0.0061642	-1.31322

2708407	**ALG3**	NM_005787	0.00616772	1.26582
3196034	**C9orf66**	NM_152569	0.00624781	-1.77628
3536396	**CGRRF1**	NM_006568	0.00635345	-1.38361
3520989	**TGDS**	NM_014305	0.00648183	-1.29708
3190242	**DNM1**	NM_004408	0.00653862	1.20565
3726691	**ABCC3**	NM_003786	0.00666346	1.21287
3474521	**SRSF9**	NM_003769.2	0.00669331	-1.32203
3565663	**DLGAP5**	NM_001146015	0.00679526	-1.22104
2693569	**ZXDC**	NM_001040653	0.00681498	1.20603
2365675	**POU2F1**	NM_002697	0.00682415	1.26972
2623388	**PARP3**	NM_001003931	0.0068525	1.31126
2736642	**PDHA2**	NM_005390	0.00689448	-1.35472
3996467	**PLXNA3**	NM_017514	0.00690298	1.2164
3428088	**ACTR6**	NM_022496	0.00699425	-1.32686
3980968	**NONO**	NM_001145408.1	0.00702538	-1.59976
4011464	**PJA1**	NM_145119	0.00705975	-1.28106
3831917	**ZNF570**	NM_144694	0.00706518	-1.34366
2466039	**ZNF692**	NM_017865	0.00711941	1.30008
3409127	**ARNTL2**	NM_020183	0.00717311	-1.23393
3434308	**SIRT4**	NM_012240	0.0072454	-1.31898
3771744	**MXRA7**	NM_001008528	0.00726019	-1.26664
3682135	**C16orf63**	NM_144600.2	0.00726615	-1.308
3452664	**P11**	NM_006025	0.00745374	-1.34318
2904528	**ZNF76**	NM_003427	0.00745477	1.24241
3379708	**MRPL21**	NM_181515	0.00749611	-1.33122
2773872	**NAAA**	NM_014435	0.00750028	1.2457
2764678	**FLJ45721**	AK127623	0.00750823	-1.20865
3215146	**NINJ1**	NM_004148	0.00757756	1.26096
3489708	**DLEU1**	NR_002605	0.00758961	-1.25501
3695916	**CENPT**	NM_025082	0.00759903	1.23671
3738205	**MRPL12**	NM_002949	0.00776853	1.27421
3419473	**RPL14**	NM_001034996.1	0.00780536	1.97944
3722917	**GRN**	NM_002087	0.00787389	1.24911
3659858	**TMEM188**	NM_153261	0.00792477	-1.52999
3883129	**MYH7B**	NM_020884	0.00793204	1.36388
3457872	**MIP**	NM_012064	0.00793296	-1.35537
4000538	**FIGF**	NM_004469	0.00798477	-1.29038
2347132	**FNBP1L**	NM_001024948	0.00805351	-1.27041
2519860	**ASNSD1**	NM_019048	0.00809028	-1.26439
3816919	**NFIC**	NM_205843	0.00809632	1.36525
3591365	**ADAL**	NM_001159280	0.00810711	-1.35085
3265952	**PNLIPRP2**	NM_005396	0.00815182	-1.42148
3805553	**RIT2**	NM_002930	0.00817236	-1.35026
3346584	**BIRC2**	NM_001166	0.00822011	-1.40785
3635159	**ST20**	NM_001100879	0.00825143	-1.6262
3468009	**ARL1**	NM_001177	0.00825155	-1.28988

2412690	**KTI12**	NM_138417	0.00828238	1.24299
2674303	**RHOA**	NM_001664.2	0.00828717	1.42995
3539147	**SNAPC1**	NM_003082	0.00829905	-1.2382
2451493	**CYB5R1**	NM_016243	0.0084029	1.24045
3641112	**FAM169B**	NM_182562	0.00842117	-1.28876
2325206	**RPL11**	NM_000975.3	0.00846021	-1.86042
3661645	**IRX6**	NM_024335	0.00846513	-1.26277
3493448	**PIBF1**	NM_006346	0.00852206	-1.37711
2831875	**SLC35A4**	NM_080670	0.00856094	1.40934
3745781	**ZNF18**	NM_144680	0.00857263	-1.20096
3844028	**RPS5**	NM_001009.3	0.00868604	-1.27343
3743502	**CTDNEP1**	NM_001143775.1	0.00869325	-1.28737
3720132	**RPL19**	NM_000981.3	0.00873373	-1.27382
3360006	**RHOG**	NM_001665	0.00876166	1.3358
3699760	**KARS**	NM_001130089.1	0.00876497	-1.33832
2954771	**GTPBP2**	NM_019096	0.00879405	1.276
2889753	**ZNF354A**	NM_005649	0.00879838	-1.47659
3045047	**RP9**	NM_203288	0.00881491	-1.40856
3702547	**COTL1**	NM_021149	0.00887136	1.3658
3008108	**LIMK1**	NM_002314	0.00889254	1.2286
2359322	**LCE3C**	NM_178434	0.0089121	-1.52035
3465593	**EEA1**	NM_003566	0.00895442	-1.31101
3633794	**ETFA**	NM_000126	0.00906635	-1.35697
3882681	**CHMP4B**	NM_176812	0.00907281	-1.60781
3969422	**RAB9A**	NM_004251	0.00908788	-1.27952
3311342	**METTL10**	NM_212554	0.00910219	-1.34524
3204061	**ENHO**	NM_198573	0.009114	-1.20732
3303255	**ERLIN1**	NM_006459	0.00921481	-1.21665
2840638	**NPM1**	NM_001037738.2	0.00926559	-1.6102
2384375	**DUSP5P**	NR_002834	0.00939706	-1.44612
2657025	**RTP4**	NM_022147	0.00947062	-1.29191
2325192	**RPL11**	NM_000975	0.00955259	-1.40953
3837464	**GLTSCR2**	NM_015710	0.00971584	1.36898
2902463	**BAT2**	NM_080686	0.00976385	1.36986
2844248	**CANX**	NM_001024649.1	0.00981573	-1.43119
3752097	**C17orf42**	NM_024683	0.00984082	-1.22054
3566949	**C14orf149**	NM_144581	0.0098536	-1.35639
3137875	**GGH**	NM_003878	0.00986981	-1.29937
3602723	**RCN2**	NM_002902	0.00987657	-1.27549
3844152	**ZNF324**	NM_014347	0.00991536	1.20254
3348911	**SDHD**	NM_003002	0.00991838	-1.58489
3009198	**RHBDD2**	NM_001040457	0.00999481	1.32359
3701297	**CDYL2**	NM_152342	0.00999576	-1.23727
3435192	**MLXIP**	NM_014938	0.0100516	1.37469
2835531	**NDST1**	NM_001543	0.0101574	1.35519
3144934	**GEM**	NM_005261	0.0103277	-1.3271

2439842	**TAGLN2**	NM_003564	0.0103993	1.22193
2924619	**TRMT11**	NM_001031712	0.0104125	-1.25029
3828162	**C19orf2**	NM_003796	0.0104538	-1.33616
2739267	**RRH**	NM_006583	0.0104685	1.21549
3623424	**COPS2**	NM_004236	0.0104689	-1.27299
3562746	**C14orf106**	NM_018353	0.0105625	-1.44846
3365437	**TSG101**	NM_006292	0.0105847	-1.37168
3111375	**TTC35**	NM_014673	0.0106337	-1.64759
2489372	**hCG_1811732**	AK125271	0.0106606	1.41025
2812120	**SDCCAG10**	NM_005869	0.0106881	-1.56265
2438093	**C1orf85**	NM_144580	0.0107323	1.27506
3538703	**MNAT1**	NM_002431	0.0107361	-1.43359
2675171	**HYAL2**	NM_033158	0.0107809	1.21827
3258260	**EXOC6**	NM_019053	0.0108413	-1.23037
2438458	**CRABP2**	NM_001878	0.0108718	1.33052
3628923	**FAM96A**	NM_032231	0.0108762	-1.38014
3755614	**STAC2**	NM_198993	0.010908	-1.29347
2951500	**TEAD3**	NM_003214	0.0109332	1.20875
2533670	**AGAP1**	NM_001037131	0.0109469	1.30523
3246182	**SLC18A3**	NM_003055	0.0110333	-1.29373
2528020	**TTLL4**	NM_014640	0.0110714	1.2317
3587457	**ARHGAP11A**	NM_014783	0.0110915	-1.24051
2888698	**LMAN2**	NM_006816	0.0110982	1.41433
3720343	**STARD3**	NM_006804	0.0111342	1.30725
3664982	**CES2**	NM_003869	0.0111427	1.2209
3041519	**TRA2A**	NM_013293	0.0113804	-1.37763
2619265	**VIPR1**	NM_004624	0.0114401	1.23824
3442282	**MLF2**	NR_026581	0.0115181	1.2661
3210179	**C9orf95**	NR_023352	0.011557	-1.34046
3759077	**SLC25A39**	NM_001143780	0.0115583	1.2574
3297536	**ANXA11**	NM_145869	0.0116289	1.20168
3475324	**TMEM120B**	NM_001080825	0.0116619	1.60665
3285614	**ZNF25**	NM_145011	0.0116624	-1.5348
2981976	**OSTCL**	NM_145303	0.0116743	1.3381
2451931	**GOLT1A**	NM_198447	0.0119206	-1.28232
3894047	**PCMTD2**	NM_018257	0.0119407	-1.20443
2438282	**IQGAP3**	NM_178229	0.0119838	1.2194
2566645	**MITD1**	NM_138798	0.0120074	-1.48736
3260423	**CUTC**	NM_015960	0.0120232	-1.45199
3263944	**PDCD4**	NM_145341	0.0120464	-1.26329
3475717	**RSRC2**	NM_198261	0.0120745	-1.34305
3248999	**REEP3**	NM_001001330	0.0120771	-1.33516
3209623	**ZFAND5**	NM_001102420	0.0121216	-1.21178
3617830	**ZNF770**	NM_014106	0.012126	-1.44875
3877265	**MACROD2**	NM_080676	0.0122187	-1.27319
2987843	**SDK1**	NM_152744	0.0124468	1.34362

3683651	**ACSM2B**	NM_182617	0.0124837	-2.9554
2903782	**ITPR3**	NM_002224	0.0124842	1.40451
3175119	**OSTF1**	NM_012383	0.0125281	-1.30104
3308397	**HSPA12A**	NM_025015	0.0126946	1.26944
2434124	**HIST2H2BE**	NM_003528	0.0127198	1.28698
2788143	**ANAPC10**	NM_014885	0.0128763	-1.52138
3625539	**NEDD4**	NM_006154	0.0129137	-1.221
4006841	**SLC9A7**	NM_032591	0.0129432	1.25401
3283920	**ARHGAP12**	NM_018287	0.0129733	-1.2442
3003228	**SUMF2**	NM_015411	0.0130728	1.2814
2402601	**UBXN11**	NM_183008	0.0131845	1.20441
2954022	**TRERF1**	NM_033502	0.0132694	1.28378
2685345	**STX19**	NM_001001850	0.013314	1.59997
3245881	**WDFY4**	NM_020945	0.0133506	-1.3953
2900091	**HIST1H2AL**	NM_003511	0.0133851	1.29769
3591044	**HAUS2**	NM_018097	0.0133939	-1.3829
3168415	**CLTA**	NM_007096	0.013474	-1.20169
3306299	**XPNPEP1**	NM_020383	0.013525	-1.38872
2617687	**XYLB**	NM_005108	0.0135407	1.28993
2953536	**TREML2**	NM_024807	0.0135765	1.42196
3592023	**B2M**	NM_004048	0.0136043	-1.32426
3839276	**NR1H2**	NM_007121	0.0136426	1.28211
3332530	**MS4A10**	NM_206893	0.013769	-1.2093
3249788	**CCAR1**	NM_018237	0.0137991	-1.22524
2904597	**PPARD**	NM_006238	0.0138104	1.43443
2361241	**ROBLD3**	NM_014017	0.0138112	-1.32929
3322251	**NUCB2**	NM_005013	0.0139173	-1.62501
2975680	**BCLAF1**	NM_014739	0.0139824	-1.26173
4017381	**TSC22D3**	NM_198057	0.0140271	-1.26384
2396817	**MTHFR**	NM_005957	0.0140847	1.25076
2680298	**MAGI1**	NM_015520	0.0141814	1.2145
2862841	**GCNT4**	NM_016591	0.0142213	1.31643
3835494	**ZNF226**	NM_001146220	0.0143248	-1.40974
2379863	**CENPF**	NM_016343	0.0144487	-1.20849
2480589	**CRIPT**	NM_014171	0.014463	-1.2166
3007438	**POM121**	NM_172020	0.0144814	1.38587
2842255	**CPLX2**	NM_006650	0.0145045	1.22129
2453793	**LAMB3**	NM_001017402	0.0145059	1.23029
3382972	**RSF1**	NM_016578	0.0145841	-1.24551
2835848	**SLC36A1**	NM_078483	0.014595	1.29206
2436338	**CRTC2**	NM_181715	0.0146828	1.23049
3644220	**NDUFB10**	NM_004548	0.0147	-1.30021
3458097	**NACA**	NM_001113203	0.0147832	1.24797
3569200	**ATP6V1D**	NM_015994	0.0147955	-1.21212
2691798	**IQCB1**	NM_001023570	0.0148401	-1.23094
3566176	**OTX2**	NM_021728	0.0148503	-1.20716

3914050	**STMN3**	NM_015894	0.0148549	-1.28477
3989089	**ZBTB33**	NM_006777	0.0149008	-1.22288
2949450	**HSPA1L**	NM_005527	0.0149143	-1.53066
3554104	**KIF26A**	NM_015656	0.0149592	-1.24051
2900750	**OR2J3**	NM_001005216	0.0149831	-1.67518
3772158	**TK1**	NM_003258	0.0152196	1.28245
2326448	**SH3BGRL3**	NM_031286	0.0152569	1.21184
2434925	**PI4KB**	NM_002651	0.0152724	1.23337
3474372	**PXN**	NM_001080855	0.0152815	1.25477
3959787	**CACNG2**	NM_006078	0.01529	-1.24097
2676671	**TKT**	NM_001135055	0.0153154	1.31629
3527418	**PARP2**	NM_005484	0.0154591	-1.28194
3504791	**EFHA1**	NM_152726	0.0154666	-1.45653
4019570	**UPF3B**	NM_080632	0.0155781	-1.42075
2674047	**LAMB2**	NM_002292	0.0155952	1.2136
2322786	**PADI1**	NM_013358	0.0156593	1.26268
4004819	**DYNLT3**	NM_006520	0.0156746	-1.51079
3424174	**MYF5**	NM_005593	0.0156844	-1.2248
3664785	**CKLF**	NM_016951	0.0156904	-1.21376
2403111	**WASF2**	NM_006990	0.0158457	1.22545
3560711	**BAZ1A**	NM_013448	0.0158607	-1.21047
2320727	**TNFRSF1B**	NM_001066	0.0159321	1.22092
3872604	**ZNF606**	NM_025027	0.0160529	-1.32583
3920816	**DSCR8**	NR_026838	0.0161162	1.21215
3434393	**DYNLL1**	NM_001037494	0.0161496	-1.81557
3865464	**OPA3**	NM_001017989	0.016267	1.21853
2624291	**PRKCD**	NM_006254	0.0163471	1.22119
2336891	**DIO1**	NM_000792	0.016415	1.33782
3148582	**EIF3E**	NM_001568	0.0164479	-1.38751
3406589	**MGST1**	NM_145791	0.0164487	-1.20198
3818376	**CLPP**	NM_006012	0.0164826	1.25284
3776139	**NDC80**	NM_006101	0.0165386	-1.27717
3063856	**GATS**	NM_178831	0.0166	1.22966
3603408	**PSMA4**	NM_002789	0.0166423	-1.21388
3688254	**PRSS8**	NM_002773	0.0167008	-1.23669
3428845	**C12orf48**	AK302724	0.016717	-1.45171
3258910	**HELLS**	NM_018063	0.0169054	-1.22989
3862471	**AKT2**	NM_001626	0.0169968	1.21755
2396461	**SRM**	NM_003132	0.0171112	1.41795
3638699	**C15orf38**	NM_182616	0.0172869	1.35396
3592511	**SQRDL**	NM_021199	0.0173972	-1.27104
2705014	**SLC7A14**	NM_020949	0.0174478	-1.21861
3952543	**SLC25A1**	NM_005984	0.0174564	1.43865
2675304	**TMEM115**	NM_007024	0.0174927	1.30322
2494749	**CNNM3**	NM_017623	0.0175323	1.31852
2732068	**SHROOM3**	NM_020859	0.0175459	1.2256

3395416	**HSPA8**	NM_006597	0.017558	1.47341
4007186	**ELK1**	NM_001114123	0.017693	1.32495
2494709	**CNNM4**	NM_020184	0.0177672	1.20477
2731381	**CXCL1**	NM_001511	0.0179251	-1.51902
2638886	**FAM162A**	NM_014367	0.0179391	-1.26211
3466284	**NDUFA12**	NM_018838	0.0179741	-1.33986
2360083	**UBAP2L**	NM_014847	0.0179802	1.23529
2674919	**MST1R**	NM_002447	0.0180509	1.34785
3134922	**PCMTD1**	NM_052937	0.0180557	-1.32088
4000704	**AP1S2**	NM_003916	0.0180779	-1.2654
2758686	**LYAR**	NM_017816	0.0181392	-1.35909
3729002	**C17orf71**	NM_018149	0.0181395	-1.31881
3311775	**DHX32**	NM_018180	0.018174	-1.26942
3282117	**ANKRD26**	NM_014915	0.0181958	-1.41309
3638204	**MFGE8**	NM_005928	0.0182936	1.25595
3708306	**ACADVL**	NM_000018	0.0183471	1.20455
3975762	**ZNF673**	NM_001129899	0.0184234	-1.27215
3183757	**RAD23B**	NM_002874	0.01845	-1.21567
3957374	**SEC14L4**	NM_174977	0.0185394	1.26417
3335327	**SSSCA1**	NM_006396	0.0189276	1.38556
3907987	**SLC13A3**	NM_022829	0.0189344	1.20233
3264621	**TCF7L2**	NM_001146274	0.0190817	1.23839
3015276	**CNPY4**	NM_152755	0.019115	1.21211
2326561	**RPS6KA1**	NM_002953	0.0192257	1.24303
3251353	**C10orf104**	NM_173473	0.0192503	-1.40368
3455516	**KRT8**	NM_002273	0.0192635	1.3785
4038494	**SETD8**	NM_020382	0.0193618	-1.30554
3655708	**C16orf53**	NM_024516	0.0194605	-1.2809
3995392	**ZNF185**	NM_007150	0.0194947	1.26081
3851911	**GADD45GIP1**	NM_052850	0.0195209	-1.26435
2473965	**C2orf18**	NM_017877	0.0195235	1.28255
3293435	**PRF1**	NM_005041	0.0195559	1.25594
3519624	**SLITRK1**	NM_052910	0.0196014	-1.38487
3991698	**HPRT1**	NM_000194	0.0196027	-1.25789
3470911	**MGC14436**	NR_026661	0.0197395	-1.28185
3921391	**WRB**	NM_004627	0.0198138	-1.29817
3608466	**MAN2A2**	NM_006122	0.0198946	1.30931
3796992	**C18orf18**	NR_026849	0.0199019	-1.41736
4026956	**HCFC1**	NM_005334	0.0200391	1.27319
2843579	**RMND5B**	NM_022762	0.0201036	1.26121
3014957	**ZNF498**	NM_145115	0.0201085	1.27236
2663551	**NUP210**	NM_024923	0.0201193	1.30894
3984779	**HNRNPH2**	NM_019597	0.0201335	-1.21495
3890272	**C20orf106**	NM_001012971	0.020193	-1.61692
3536336	**CDKN3**	NM_005192	0.0202454	-1.29681
3860491	**ZNF260**	NM_001012756	0.0203274	-1.2406

3509885	**ALG5**	NM_013338	0.0204333	-1.39266
3407793	**PYROXD1**	NM_024854	0.0204672	-1.67358
3996404	**GDI1**	NM_001493	0.0205397	1.30152
3471224	**GPN3**	NM_016301	0.0205409	-1.33684
3980981	**ITGB1BP2**	NM_012278	0.0205595	1.27666
3202136	**C9orf82**	BC071953	0.0205734	-1.23535
3216969	**XPA**	NR_027302	0.0205777	-1.30994
2947889	**GABBR1**	NM_001470	0.0205866	1.24433
3109191	**POLR2K**	NM_005034	0.0206005	-1.29156
2421782	**CCBL2**	NM_001008661	0.0206517	-1.29672
3976639	**PORCN**	NM_022825	0.0207674	1.2955
3565571	**WDHD1**	NM_007086	0.0207722	-1.21004
2833924	**SH3RF2**	NM_152550	0.0208233	1.44449
3653000	**UBFD1**	NM_019116	0.0208427	-1.21497
3421523	**YEATS4**	NM_006530	0.0209241	-1.52697
2465728	**OR2B11**	NM_001004492	0.0209332	-1.45181
4016428	**BEX2**	NM_032621	0.0209731	-1.24462
3337835	**IGHMBP2**	NM_002180	0.0211482	-1.28495
3617170	**AVEN**	NM_020371	0.0211643	-1.36229
2680591	**LRIG1**	NM_015541	0.0211891	1.31424
3344861	**C11orf54**	AK292215	0.0212401	-1.29186
2459924	**ABCB10**	NM_012089	0.0212924	-1.29222
2397948	**EPHA2**	NM_004431	0.0213082	1.28029
2452754	**SLC26A9**	NM_052934	0.0213621	1.3707
3909395	**DPM1**	NM_003859	0.0213924	-1.38114
3868828	**KLK10**	NM_002776	0.021394	1.27399
2693014	**SLC12A8**	NM_024628	0.0214515	1.3045
2711751	**TMEM44**	NM_138399	0.0214554	1.23462
3536905	**KTN1**	NM_182926	0.0214981	-1.26463
2948564	**MDC1**	NM_014641	0.0215196	1.23586
3453120	**ZNF641**	NM_152320	0.0215646	-1.2112
3839006	**PTOV1**	NM_017432	0.0216546	1.27723
3550485	**VRK1**	NM_003384	0.0217487	-1.3131
3330943	**OR8K1**	NM_001002907	0.0218046	-1.28651
3077573	**ARHGEF5L**	NM_001003702	0.021862	-1.23428
3757329	**JUP**	NM_002230	0.0220004	1.35904
3869784	**ZNF28**	NM_006969	0.0220078	-1.49625
3257031	**STAMBPL1**	NM_020799	0.0220482	-1.23245
3319613	**RPL27A**	NM_000990	0.0220895	1.24725
3915087	**USP25**	NM_013396	0.0221055	-1.23093
3300793	**C10orf4**	NM_145246	0.0221252	-1.44928
3139950	**LACTB2**	NM_016027	0.0221483	-1.5687
2322103	**SPEN**	NM_015001	0.0221993	1.23243
3304385	**C10orf95**	BC126459	0.0222302	-1.37339
3646542	**ALG1**	NM_019109	0.0222455	1.49366
3462843	**NAP1L1**	NM_004537	0.0223433	-1.51539

3105467	**E2F5**	NM_001951	0.0223844	-1.26918
3928587	**KRTAP21-2**	NM_181617	0.0224607	-1.75648
3816699	**ZNF57**	NM_173480	0.0225075	-1.25758
3819374	**CCL25**	NM_005624	0.0225387	-1.27726
3800619	**ROCK1**	NM_005406	0.0226313	-1.34421
3345774	**JRKL**	NM_003772	0.0228398	-1.25814
2532894	**DGKD**	NM_152879	0.0228573	1.26746
3719112	**ZNHIT3**	NM_004773	0.0228592	-1.32646
3961042	**FLJ23865**	AK074445	0.0229813	-1.45149
3290649	**FAM13C**	NM_001143773	0.0230358	-1.22229
3626704	**SLTM**	NM_024755	0.0230447	-1.45058
3164086	**ADAMTSL1**	NM_001040272	0.0230954	-1.27235
2934308	**IGF2R**	NM_000876	0.0231106	1.22723
3089102	**EPB49**	NM_001978	0.0231818	1.20756
3651478	**ACSM3**	NM_005622	0.0232591	-1.24592
4045643	**S100A16**	NM_080388	0.0233032	1.23745
3444472	**TAS2R50**	NM_176890	0.0233914	2.96915
3761378	**HOXB5**	NM_002147	0.0234231	1.27715
3971768	**PRDX4**	NM_006406	0.0234441	-1.28535
2899233	**HIST1H3E**	NM_003532	0.0236109	1.20146
2474341	**CAD**	NM_004341	0.0236861	1.33805
3914181	**UCKL1**	NM_017859	0.0238025	1.23085
3996083	**TMEM187**	NM_003492	0.0239496	-1.25304
3463522	**PAWR**	NM_002583	0.0240481	-1.29544
3722535	**ARL4D**	NM_001661	0.0240746	-1.21445
3258168	**KIF11**	NM_004523	0.0240974	-1.2843
2358360	**ECM1**	NM_004425	0.0242617	1.2693
3567187	**DHRS7**	NM_016029	0.0242844	-1.30511
3696554	**TMED6**	NM_144676	0.0243984	1.21077
3251926	**KIAA0913**	NM_015037	0.0244411	1.32636
3269280	**FAM175B**	NM_032182	0.0244818	-1.22189
2439944	**PIGM**	NM_145167	0.0244975	1.30715
2329041	**KIAA1522**	NM_020888	0.0245579	1.363
3662139	**MT1E**	NM_175617	0.0245638	-1.40818
3645253	**SRRM2**	NM_016333	0.0246528	1.20863
3740201	**MYO1C**	NM_001080779	0.0246819	1.25895
2374746	**NAV1**	NM_020443	0.0248039	1.31087
3504760	**ZDHHC20**	NM_153251.3	0.0249967	-1.32794
